# Upregulation of *Hsp27* via further inhibition of histone *H2A* ubiquitination confers protection against myocardial ischemia/reperfusion injury by promoting glycolysis and enhancing mitochondrial function

**DOI:** 10.1038/s41420-023-01762-x

**Published:** 2023-12-19

**Authors:** Pilong Shi, Jiawei Wu, Minghui Li, Yonggang Cao, Jiabi Wu, Ping Ren, Kai Liu, Jiajun Zhou, Yuetong Sha, Qianhui Zhang, Hongli Sun

**Affiliations:** 1 Department of Pharmacology, Harbin Medical University-Daqing, Daqing, Heilongjiang 163319 China; 2 Department of Pharmaceutics, Harbin Medical University-Daqing, Daqing, Heilongjiang 163319 China

**Keywords:** Myocardial infarction, Metabolomics

## Abstract

Research suggests that ischemic glycolysis improves myocardial tolerance to anoxia and low-flow ischemia. The rate of glycolysis during ischemia reflects the severity of the injury caused by ischemia and subsequent functional recovery following reperfusion. Histone *H2AK119* ubiquitination (*H2Aub*) is a common modification that is primarily associated with gene silencing. Recent studies have demonstrated that *H2Aub* contributes to the development of cardiovascular diseases. However, the underlying mechanism remains unclear. This study identified *Hsp27* (heat shock protein 27) as a *H2Aub* binding protein and explored its involvement in mediating glycolysis and mitochondrial function. Functional studies revealed that inhibition of *PRC1* (polycomb repressive complex 1) decreased *H2Aub* occupancy and promoted *Hsp27* expression through inhibiting ubiquitination. Additionally, it increased glycolysis by activating the *NF-κB*/*PFKFB3* signaling pathway during myocardial ischemia. Furthermore, *Hsp27* reduced mitochondrial ROS production by chaperoning *COQ9*, and suppressed ferroptosis during reperfusion. A delivery system was developed based on PCL-PEG-MAL (PPM)-PCM-SH (CWLSEAGPVVTVRALRGTGSW) to deliver PRT4165 (PRT), a potent inhibitor of *PRC1*, to damaged myocardium, resulting in decreased *H2Aub*. These findings revealed a novel epigenetic mechanism connecting glycolysis and ferroptosis in protecting the myocardium against ischemia/reperfusion injury.

## Introduction

Rapid advancements in reperfusion treatments have revolutionized the management of myocardial infarction; however, it remains a significant global cause of mortality [1]. Thrombolytic treatment or percutaneous coronary intervention is the preferred treatment for blocked arterial revascularization in patients of myocardial infarction. However, reperfusion can cause severe injury to the myocardium via inflammatory mediators and oxidative stress, commonly referred to as myocardial ischemia/reperfusion injury (MI/RI) [2]. Moreover, recent evidence suggests that disorders of energy metabolism play a key role in the pathogenesis of MI/RI and promote cardiomyocyte injury and myocardial dysfunction [3, 4]. Therefore, improving the energy metabolism of cardiomyocytes is of the utmost importance for mitigating MI/RI.

Glycolysis is a conserved and ancient metabolic pathway that predates the presence of atmospheric oxygen. In contrast to mitochondrial oxidative phosphorylation, tumor cells exhibit aerobic glycolysis, also known as the “Warburg effect.” This distinct metabolic behavior enables tumor cells to generate ATP and energy in an oxygen-independent manner [5]. This metabolic adaptation enables tumor cells to survive in oxygen-deprived environments and to rapidly generate ATP. Evidence suggests that ischemic glycolysis improves myocardial tolerance to anoxia and low-flow ischemia. *GLUT4*, a crucial regulator of enhanced glycolysis during ischemia, has been established as a significant protective factor against ischemic injury [6]. Pinocembrin, a natural cardioprotective compound, has been shown to alleviate acute ischemia-induced myocardial injury through the promotion of glycolysis [7]. Beltran *et al*. demonstrated that glycolysis confers protection against MI/RI by decreasing ROS production [8]. Recent studies have suggested that m6A demethylase, fat mass- and obesity-associated protein (*FTO*), attenuates cardiac dysfunction in mice by regulating glycolysis and glucose uptake [9]. However, several potential mechanisms related to glycolysis in the context of MI/RI have not been identified.

Molecular chaperones such as heat shock proteins (*HSPs*) play a pivotal role in preventing protein misfolding and aggregation, thereby maintaining protein homeostasis [10, 11]. *HSPs* facilitate the adaptation of cells to their surroundings and enhance their survival under lethal conditions [12]. *HSPs* can increase cellular resistance to stressors, including heat shock, oxidative conditions, cytotoxic drug exposure, and apoptosis-inducing factors [13]. *Hsp27*, also known as *HSPB1*, is a ubiquitous small heat shock protein expressed abundantly in the nucleus and cytoplasm [14]. It is essential for maintaining protein homeostasis, particularly during periods of stress [15]. Research has shown that *Hsp27* provides cardioprotection against oxidative stress, apoptosis, and infarction during hypoxic stress or ischemia-induced myocardial injury [12]. Additionally, levodopa treatment has been found to induce cardioprotective effects by increasing *Hsp27* activity [16]. *Hsp27* overexpression protects against ischemia/reperfusion-induced cardiac dysfunction [17]. Although the cardioprotective role of *Hsp27* is well-established, the underlying mechanisms remain poorly understood.

Eukaryotic genomic DNA forms chromatin structures through its interaction with histone and non-histone proteins [18], including post-translational histone modifications, such as phosphorylation, ubiquitination, and acetylation, which regulate chromatin dynamics and mediate chromatin-based nuclear processes [19, 20]. Histone *H2A* ubiquitination, which occurs on lysine residues K129 and K15, but primarily on *H2AK119* (abbreviated as *H2Aub*), is a prevalent modification with 5-15% *H2Aub* occurring in mammalian cells [21, 22]. Current research suggests that *H2Aub* is involved in maintaining genomic stability, DNA repair, and suppression of specific transcriptional programs, whereas *H2A* deubiquitination is correlated with genomic instability, activating transcription, promoting target gene activation, and facilitating cell-cycle transition [23, 24]. The ubiquitin *E3* ligase responsible for *H2A* ubiquitination is polycomb repressive complex 1 (*PRC1)* [25], the core components of which are *BMI1, RING1A*, and *RING1B*, with *RING1B* serving as the catalytic protein. *PRC1*-associated *E3* ligase activity is modulated at multiple stages, and *RING1B* self-ubiquitination is crucial for its catalytic activity [26, 27]. Recent evidence suggests that *H2Aub* and its ubiquitinating enzyme *PRC1* are responsible for the onset and progression of cardiovascular disorders [28, 29]. Although researchers are primarily investigating the relationship between *H2Aub* and tumors, the role of *PRC1* in regulating *H2Aub* levels in chromatin and the involvement of transcriptional targets in cardiovascular disorders, specifically MI/RI, is yet to be determined.

This study established a strong association between *H2Aub* chromatin and the enrichment of *Hsp27*, suggesting an interaction between these two factors. Moreover, *Hsp27* knockdown significantly downregulated *PFKFB3* expression and worsened hypoxia-induced myocardial cell injury, suggesting that *H2Aub* is at least partially responsible for *Hsp27*-mediated glycolytic alterations in hypoxic cardiomyocytes. Additional research confirmed that *Hsp27* maintained mitochondrial function in hypoxic-reoxygenated (H-R) cardiomyocytes by stabilizing *COQ9*. PCM-SH, a 21-mer peptide isolated through phage display, exhibited a 180-fold higher binding affinity for primary cardiomyocytes than the control, suggesting high specificity [30]. Based on this mechanism, PPMP-coated PRT (PRT@PPMP) nanoparticles were developed to inhibit *PRC1*-mediated *H2Aub* with the goal of further increasing glycolysis levels, improving mitochondrial function, and inhibiting MI/RI. This study suggests that the targeted manipulation of *H2A*ub levels could be a promising therapeutic strategy for MI/RI.

## Results

### *Hsp27* is upregulated after hypoxia and enhances glycolysis in cardiomyocytes

The literature suggests that glycolysis occurs in MI [8]; however, the underlying mechanistic details, particularly regarding the metabolic reprogramming responses to ischemia in cardiomyocytes, are not fully understood. To establish the MI model in rats, myocardial ischemia was induced for 30 min, and electrocardiograms were monitored, which revealed significant ST-segment elevation in MI rats (Fig. S1A). Echocardiography results indicated a significant decrease in left ventricular fractional shortening (LVFS) and left ventricular ejection fraction (LVEF) in MI rats (Fig. S1B–D). Successful establishment of the MI model was confirmed by hematoxylin and eosin (HE) staining, *H2AX* immunofluorescence, and triphenyltetrazolium chloride (TTC) staining (Fig. S1E–G). ATP and secreted lactate levels were measured in the myocardial tissue, revealing a metabolic shift from oxidative phosphorylation to glycolysis due to ischemia, which resulted in reduced ATP levels and increased lactate secretion (Fig. S1H–I). Metabolic profiling confirmed the accumulation of glycolytic intermediates in ischemic rats (Fig. S1J). In vitro study, hypoxia was induced for 12 h in cardiomyocytes, and CCK-8 assay results demonstrated that hypoxia significantly reduced the viability of myocardial cells (Fig. S2A-B). ATP and lactate levels were measured to investigate glycolytic alterations under hypoxic conditions, revealing that hypoxia decreased ATP content and promoted lactate secretion (Fig. S2C-D). Additionally, Seahorse XF assays measuring the extracellular acidification rate (ECAR) indicated that hypoxia increased glycolysis in the myocardial cells (Fig. S2E-F).

**Fig. 1:**
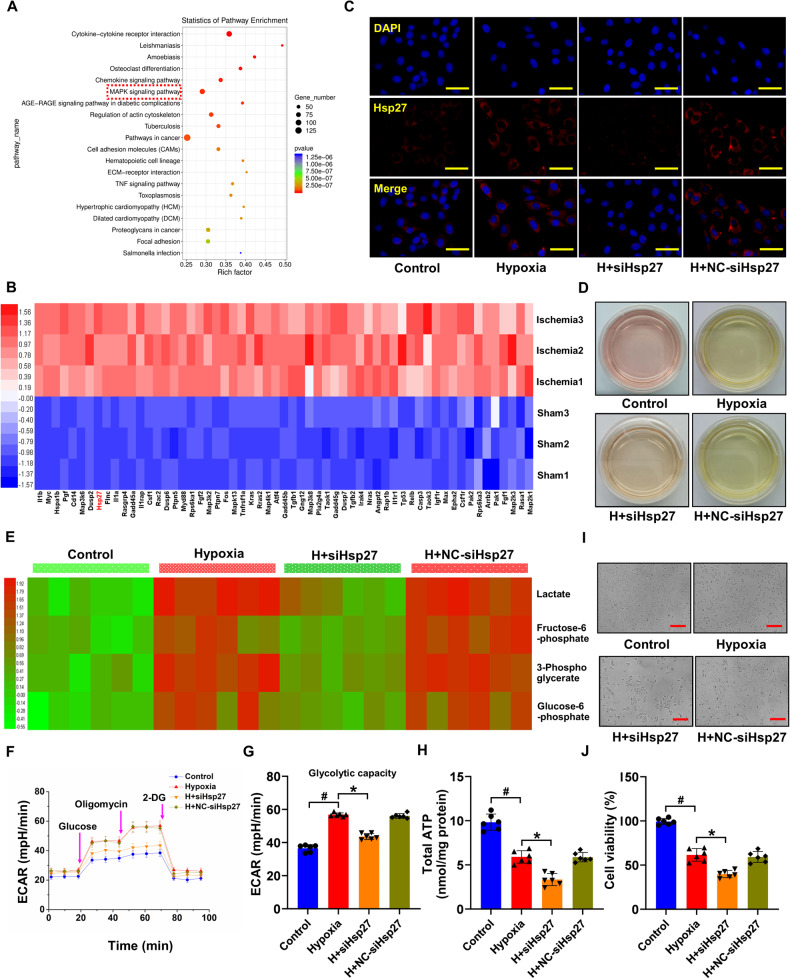
*Hsp27* knockdown inhibits glycolysis in hypoxic cardiomyocytes. **A** Pathway enrichment statistics for the identified mRNA. **B** Heat map showing upregulated mRNA of the *MAPK* signaling pathway in the rat heart after 30 min of ischemia. Color-coded representation of gene expression levels: Blue and red represented low and high expression, respectively. Data are representative of three independent experiments. **C** Immunofluorescence images of *Hsp27* (red) staining in cardiomyocytes cultured with or without *Hsp27* knockdown after 12 h of hypoxia. **D** Culture medium acidification following *Hsp27* depletion in 12-h hypoxic cardiomyocytes. **E** Heat map showing glycolytic intermediates 12 h after hypoxia stimulation. Color-coded representation of gene expression levels: Green and red represented low and high expression, respectively. **F**–**G** Graphs showing the results of glycolytic stress analyses using a Seahorse XF analyzer to assess the response of cardiomyocytes to oligomycin, glucose, and 2-DG. **H** Intracellular ATP levels in cardiomyocytes. **I**–**J** Detection of cardiomyocyte viability. Data are presented as mean ± SD (n = 6). ^#^*P* < 0.05 vs. Control group; ^*^P < 0.05 vs. Hypoxia group. Scale bars: (**C**) 50 μm; (**I**) 100 μm.

RNA sequencing was performed in rats with and without MI to identify differentially expressed genes and signaling pathways. The results revealed that activation of the *MAPK* signaling pathway, which promotes glycolysis [31, 32], was associated with MI (Fig. 1A). Furthermore, a subset of ten significantly upregulated genes (log2 fold-change >2 and *P* < 0.01) in the *MAPK* signaling pathway was selected (Fig. 1B). qRT-PCR was used to validate changes in gene expression after hypoxia in vitro and in vivo, revealing significant increases in the expression of *Il1b*, *Myc*, *Hspa1b*, *Pgf*, *Cd14*, *Map3k6*, *Dusp2*, *Hsp27*, *Flnc*, and *Il1α* in the myocardial tissue of MI rats and hypoxic cardiomyocytes (Fig. S3A-B). The role of these genes in glycolysis was determined by individual gene knockdown in cardiomyocytes, revealing that only *Hsp27* depletion significantly decreased acidification of the culture medium (Fig. S3C, Fig. 1C-D). Additionally, alterations in *Hsp27* expression were detected in fibroblasts and endothelial cells, with more pronounced changes in cardiomyocytes (Fig. S4A). Metabolic profiling revealed increased production of glycolytic intermediates in hypoxic cardiomyocytes, which was reduced upon *Hsp27* knockdown (Fig. 1E). Furthermore, *Hsp27* knockdown reduced glycolytic flux in cardiomyocytes (Fig. 1F-G) and decreased cellular ATP production (Fig. 1H). Investigation of cardiomyocyte viability using CCK-8 revealed that *Hsp27* knockdown worsened hypoxia-induced damage to cardiomyocytes by reducing their glycolytic capacity (Fig. 1I-J). These findings suggested the involvement of *Hsp27* in glycolysis of hypoxic cardiomyocytes.

### *Hsp27* enhances glycolysis by activating *PFKFB3* transcription

**Fig. 2:**
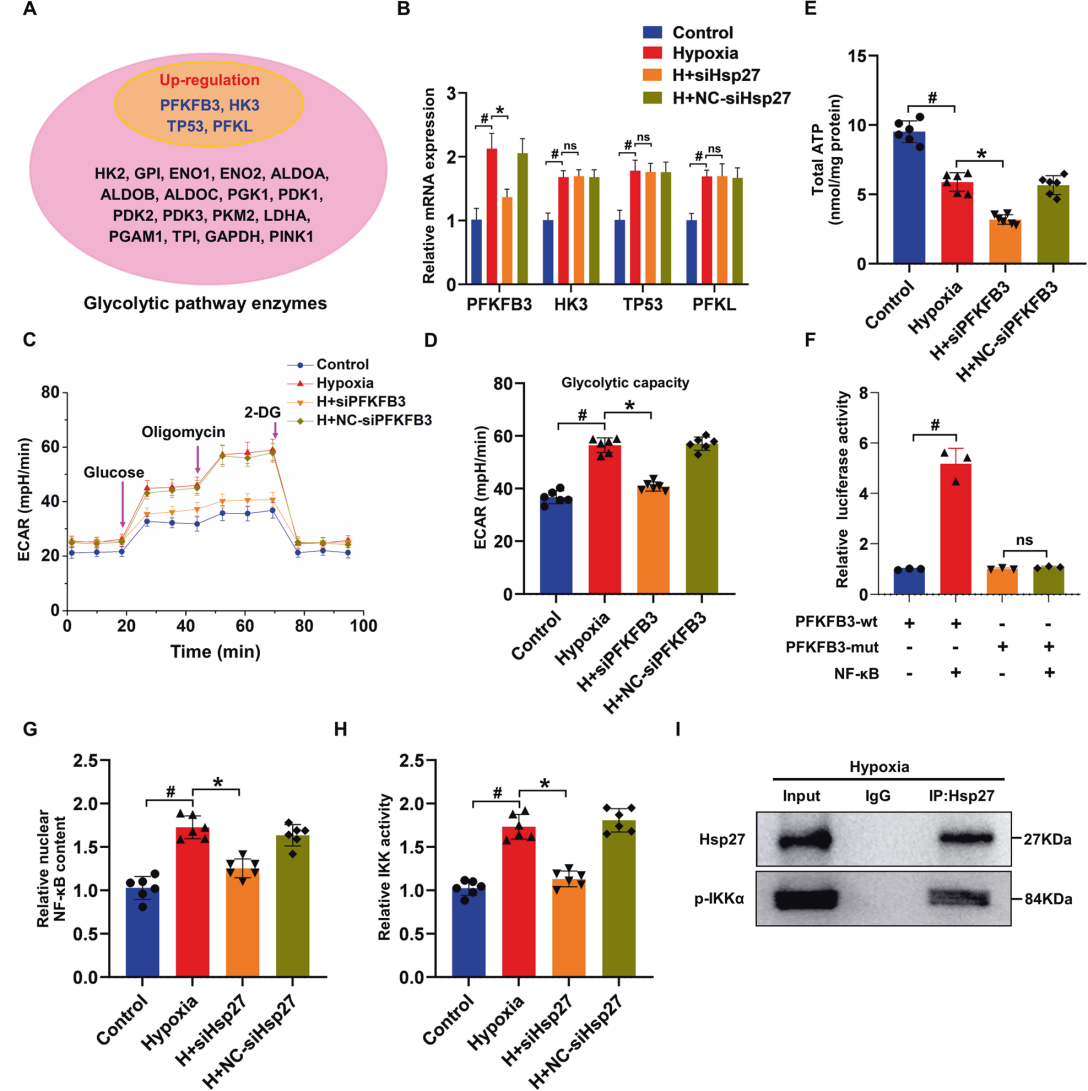
*Hsp27* promotes glycolysis by regulating *PFKFB3* via *NF-κB*. **A** Schematic representation of upregulated glycolytic pathway enzymes. **B** Relative mRNA expression levels of *PFKFB3*, *HK3*, *TP53*, and *PFKL* detected using qRT-PCR in *Hsp27* knockdown cardiomyocytes after 12 h of culture under hypoxic conditions. **C**, **D** Seahorse XF assay measuring the ECAR of cardiomyocytes cultured with or without *PFKFB3* knockdown under control and hypoxic conditions for 12 h. **E** ATP levels in the cardiomyocytes. **F** Luciferase activity analysis in cells following co-transfection with luciferase reporters containing the *NF-κB* mimic and *PFKFB3*-wt. Data are presented as mean ± SD (n = 3). ^#^*P* < 0.05 vs. the *PFKFB3*-wt group. **G** Statistical analysis of relative nuclear *NF-κB* content in cardiomyocytes. **H** Detection of *IKK* activity in cardiomyocytes cultured with or without *Hsp27* knockdown and subjected to hypoxia for 12 h. **I** Immunoprecipitation using control IgG or anti-*Hsp27* in 12-h hypoxic cardiomyocytes. Immunoblotting of precipitates for *p-IKKα* and *Hsp27*. Data are presented as mean ± SD (n = 6). ^#^*P* < 0.05 vs. Control group; ^*^P < 0.05 vs. Hypoxia group; ns, not significant.

The initial concern was whether changes in the expression of glycolytic pathway enzymes could explain the alterations in glycolytic flux observed during MI. RNA-seq analyses of these pathway enzymes revealed an increase in *PFKFB3*, *HK3*, *TP53*, and *PFKL* expression in MI rats (Fig. 2A). Analysis of the mRNA levels of *PFKFB3*, *HK3*, *TP53*, and *PFKL* in cardiomyocytes revealed that *PFKFB3* expression was decreased upon *Hsp27* knockdown, whereas the levels of *HK3*, *TP53*, and *PFKL* remained unchanged (Fig. 2B). Subsequently, *PFKFB3* blockade was found to suppress glycolysis in cardiomyocytes. Figure 2C–E demonstrated that suppressing *PFKFB3* caused a significant decline in ATP production and glycolytic capacity in hypoxic cardiomyocytes.

Previous studies have highlighted that *NF-κB* activation may increase *PFKFB3* expression [33]. To confirm direct binding between *PFKFB3* and *NF-κB*, a luciferase reporter assay was performed using wild-type and mutant plasmids of *PFKFB3*. Co-transfection of luciferase plasmids with *NF-κB* mimic/NC into cells revealed that the *NF-κB* mimic significantly increased luciferase activity in the wild-type *PFKFB3* plasmid, but did not affect the mutant *PFKFB3* luciferase activity (Fig. 2F). *NF-κB* typically forms a heterodimer with P65 and P50 and is rendered inactive in the cytoplasm due to binding to the inhibitory protein *IκB* [34, 35], resulting in the formation of a trimer complex. Nuclear *NF-κB* content was increased in response to hypoxia, but this effect was reversed when *Hsp27* was knocked down (Fig. 2G). These data suggests that *Hsp27* may affect *NF-κB* activation in hypoxic cardiomyocytes. To further validate this hypothesis, *IKK* activity was assessed, and the results indicated that hypoxia promoted *IKK* activity, which was subsequently reversed by *Hsp27* knockdown (Fig. 2H). The presence of *p-IKKα* in the immunoprecipitates was consistent with the high tendency of *NF-κB* activation in cardiomyocytes (Fig. 2I). These findings suggested that *Hsp27* promoted *PFKFB3* transcription under hypoxic conditions by binding and activating *IKK*.

### *Hsp27* improves mitochondrial function in H-R cardiomyocytes

**Fig. 3:**
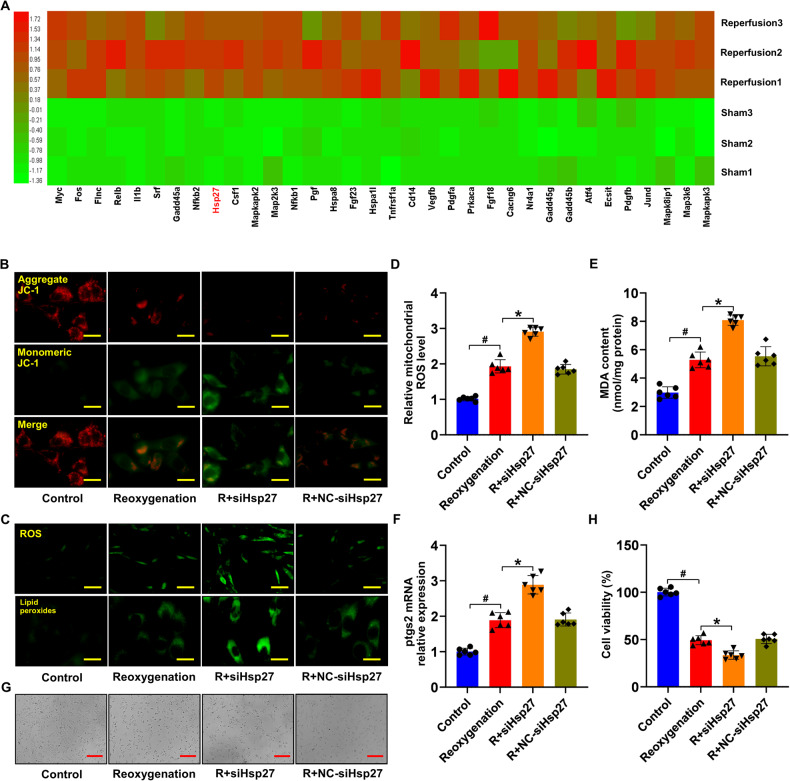
*Hsp27* improves mitochondrial function in hypoxic-reoxygenated cardiomyocytes. **A**, **B** Heat map depicting differentially expressed genes of the *MAPK* signaling pathway in cardiac tissue between the sham and MI/RI groups. Color-coded representation of gene expression levels: Green and red represented low and high expression, respectively. Data are representative of three independent experiments. **B** Representative images of JC-1 fluorescence in the H-R cardiomyocytes. **C** Detection of cellular ROS and lipid peroxide levels in the cardiomyocytes. **D** Statistical analysis of mitochondrial ROS levels. **E** Cellular MDA levels in cardiomyocytes. **F** Relative mRNA levels of *ptgs2* in cardiomyocytes determined using qRT-PCR. **G**, **H** Viability of cardiomyocytes in the different groups. Data are presented as mean ± SD (n = 6). ^#^*P* < 0.05 vs. Control group; ^*^P < 0.05 vs. Reoxygenation group. Scale bars: [**C** (top)] 50 μm; [**B**, **C** (bottom)] 25 μm; and (**G**) 100 μm.

To investigate the potential protective effects of *Hsp27* during reperfusion, RNA sequencing was performed in rats with or without MI/RI. Interestingly, *Hsp27* expression remained high in the MI/RI model (Fig. 3A). Literature indicates that *Hsp27* maintains cell membrane integrity under various stresses [36], providing cardiac protection via the chaperone effect and facilitating cytoskeleton reconstruction under stress. *Hsp27* also induces antioxidant mechanisms through the association of myocardial lesion generation with free radical production [37–39]. Furthermore, *Hsp27* significantly inhibits ROS generation [40, 41]. Mitochondria are the primary source of ROS production in cardiomyocytes [42]. Mitochondrial membrane potential is commonly used to assess mitochondrial function, and a decrease in the mitochondrial membrane potential suggests mitochondrial dysfunction. Therefore, to confirm the significance of *Hsp27* in improving mitochondrial function, the mitochondrial membrane potential was evaluated in H-R cardiomyocytes by *Hsp27* knockdown. H-R cardiomyocytes exhibited significantly higher JC-1 monomer levels than the control cardiomyocytes. Moreover, *Hsp27* knockdown further increased JC-1 monomer levels compared to those in H-R cardiomyocytes (Fig. 3B). To investigate the association between *Hsp27* and H-R-induced ferroptosis, we assessed ROS levels, lipid peroxides, mitochondrial ROS, MDA content, and *ptgs2* mRNA expression and found that *Hsp27* knockdown increased these parameters in H-R cardiomyocytes (Fig. 3C–F). *Hsp27* silencing further reduced the viability of H-R cardiomyocytes (Fig. 3G–H). These findings suggested that *Hsp27* mitigated mitochondrial dysfunction and ferroptosis in H-R cardiomyocytes.

### *Hsp27* functions as a chaperone of *COQ9* in H-R cardiomyocytes

**Fig. 4:**
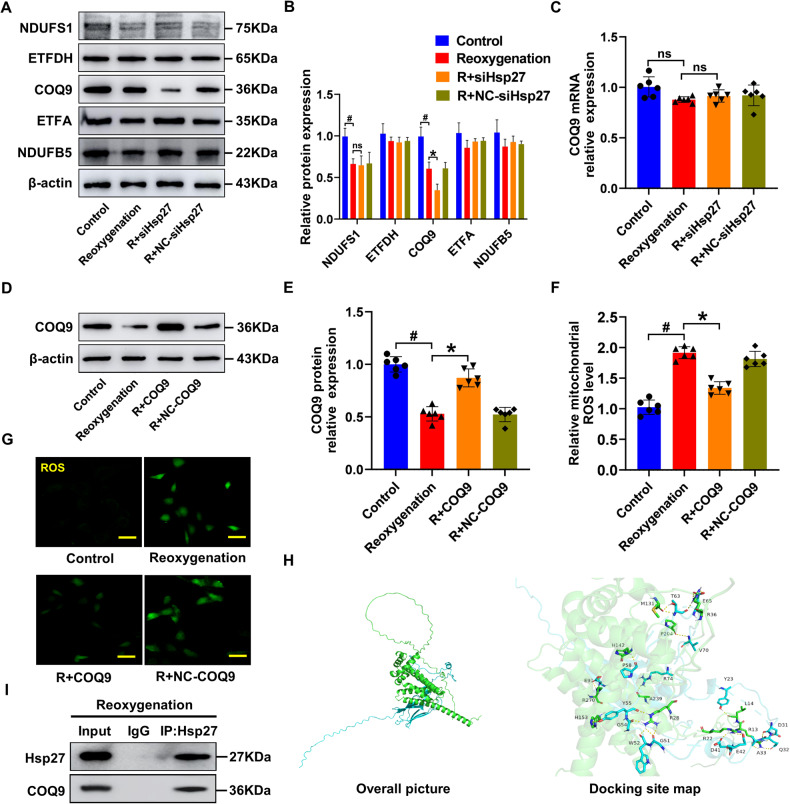
*Hsp27* stabilizes *COQ9* levels in hypoxic-reoxygenated cardiomyocytes. **A**, **B** Western blot analysis of *NDUFS1*, *ETFDH*, *COQ9*, *ETFA*, and *NDUFB5* in H-R cardiomyocytes. **C** Relative mRNA expression levels of *COQ9* detected using qRT-PCR in *Hsp27* knockdown cardiomyocytes. **D**, **E** Immunoblot analysis of *COQ9* in cardiomyocytes transfected with *COQ9*. **F** Statistical analysis of mitochondrial ROS levels. **G** Detection of cellular ROS levels in H-R cardiomyocytes. **H** Diagram showing the binding site of *Hsp27* (blue) and *COQ9* (green). **I** Immunoprecipitation using control IgG or anti-*Hsp27* in H-R cardiomyocytes. Immunoblotting of precipitates for *COQ9* and *Hsp27*. Data are presented as mean ± SD (n = 6). ^#^*P* < 0.05 vs. Control group; ^*^P < 0.05 vs. Reoxygenation group; ns, not significant. Scale bars: 50 μm.

The mitochondrial electron transport chain (ETC) uses a series of electron transfer reactions to generate ATP via oxidative phosphorylation. The generation of ROS during electron transfer can lead to oxidative stress and homeostatic signaling during disease pathology [43]. Thus, the activity of ETC complex I, whose dysfunction is primarily responsible for mitochondrial ROS production [44], was detected in this study. The results indicated that *Hsp27* knockdown resulted in a further reduction in *COQ9* protein expression compared to *NDUFS1*, *ETFDH*, *ETFA*, and *NDUFB5* (Fig. 4A, B). However, the mRNA levels of *COQ9* were not altered by *Hsp27* knockdown (Fig. 4C). The reduction in *COQ9* protein levels was independent of alterations in mRNA levels, excluding the possibility of transcriptional modifications. To investigate the effect of *COQ9* on mitochondrial ROS production, we overexpressed *COQ9* in H-R cardiomyocytes and observed a significant reduction in both mitochondrial and cellular ROS levels following *COQ9* overexpression (Fig. 4D, G). Researchers have found that small *HSPs* function as molecular chaperones in the mitochondrial intermembrane space, and that the mitochondrial interactome of *Hsp27* is enriched in transmembrane proteins of the inner mitochondrial membrane [45]. Therefore, we explored the potential role of *Hsp27* as a molecular chaperone in maintaining *COQ9* expression using Cluspro software, and the results confirmed a significant binding interaction between *Hsp27* and *COQ9* (Fig. 4H). *COQ9* was detected in the immunoprecipitates, validating the prediction of the Cluspro software (Fig. 4I). These data showed that *Hsp27* acted as a chaperone for *COQ9* in H-R cardiomyocytes.

### *Hsp27* has been identified as a *H2Aub* nucleosome-associated gene in cardiomyocytes

**Fig. 5:**
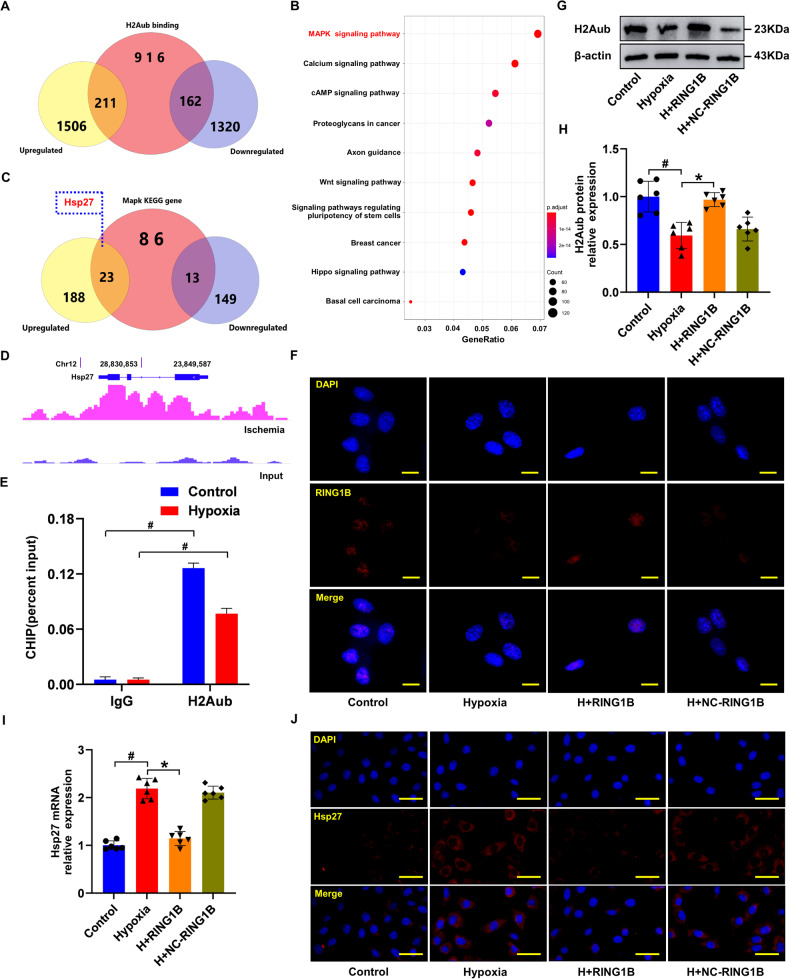
*RING1B* increases *H2Aub* occupancy of *Hsp27*. **A** Venn diagram illustrating the overlap of 1,289 genes with *H2Aub* binding and 211 upregulated genes. **B** KEGG pathway analysis of ChIP-seq data. **C** Venn diagram illustrating the overlap of 122 genes involved in the *MAPK* signaling pathway with *H2Aub* binding and 211 upregulated genes. **D** *H2Aub* ChIP–seq occupancy profiles at the *Hsp27* loci in ischemic myocardial tissue. **E** ChIP–qPCR validation of *H2Aub* binding to *Hsp27* in normal and hypoxic cardiomyocytes. Data are presented as mean ± SD, n = 6 independent replicates. ^#^*P* < 0.05 vs. IgG group. **F** Immunofluorescence analysis of *RING1B* in cardiomyocytes treated with hypoxia for 12 h with or without *RING1B* or control vector (NC-*RING1B*) transfection. **G**, **H** Immunoblot analysis of *H2Aub* in hypoxic cardiomyocytes, with or without *RING1B* or control vector (NC-*RING1B*) transfection. **I** Quantification of *Hsp27* expression in cardiomyocytes using qRT-PCR. **J** Representative images of *Hsp27* staining (red) in cardiomyocytes counterstained with DAPI (blue). Data are presented as mean ± SD (n = 6). ^#^*P* < 0.05 vs. Control group; ^*^P < 0.05 vs. Hypoxia group. Scale bars: (**F**) 25 μm; (**J**) 50 μm.

The role of *H2Aub* was not well understood until recent studies revealed its association with DNA damage repair and gene suppression [46, 47]. *H2Aub* ChIP-seq analyses were conducted to identify *H2Aub* binding genes, and determine the corresponding transcriptional alterations in the genome of MI rats. Integration of *H2Aub* RNA-seq and ChIP-seq datasets facilitated the identification of 162 downregulated and 211 upregulated genes (Fig. 5A). Interestingly, KEGG analysis revealed the enrichment of *H2Aub*-regulated genes in the *MAPK* signaling pathway (Fig. 5B). In particular, the Venn diagram revealed that 23 genes were involved in the *MAPK* signaling pathway among the 211 upregulated genes with *H2Aub* occupancy. Additionally, *Hsp27* was among the 23 upregulated genes with *H2Aub* occupancy (Fig. 5C). ChIP–seq of *H2Aub* revealed that ischemia increased *H2Aub* occupancy within *Hsp27* gene (Fig. 5D). The ChIP-qPCR results also confirmed *H2Aub* binding to *Hsp27* in both normal and hypoxic cardiomyocytes (Fig. 5E). Overexpression of *RING1B* in hypoxic cardiomyocytes increased *H2Aub* protein expression, but decreased the mRNA and protein levels of *Hsp27* (Fig. 5F–J). These findings suggested the need for further mechanistic studies on *H2Aub*-regulated *Hsp27*, and indicated that *RING1B*-mediated *H2Aub* on *Hsp27* inhibited transcriptional initiation.

### Design, synthesis, and characterization of PRT@PPMP

**Fig. 6:**
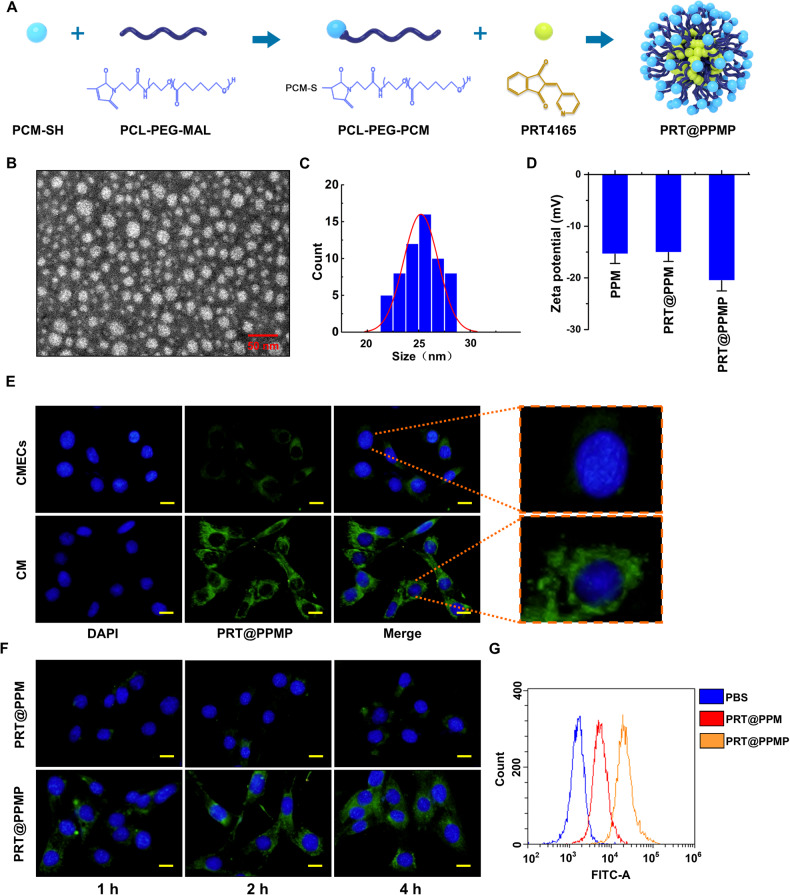
Preparation and characterization of PRT@PPMP. **A** Schematic illustrating the design of the PRT@PPMP. **B** Electron microscopic images of PRT@PPMP. **C** Size distribution. **D** The surface charge of PPM, PRT@PPM, and PRT@PPMP determined using the zeta potential and dynamic light scattering. **E** Fluorescence microscopy revealing the cellular uptake of PRT@PPMP by CMECs and CMs. **F** Immunofluorescence images showing intracellular localization of PRT@PPM and PRT@PPMP within CMs at different time points. Green-labeled PRT@PPM and PRT@PPMP with DAPI-stained nuclei (blue). **G** Cellular uptake of PRT@PPM and PRT@PPMP by CMs, observed through flow cytometry analysis. Data are presented as mean ± SD (n = 6). Scale bars: 25 μm.

PRT effectively inhibits *PRC1*-mediated *H2Aub*, and if nanocomplexes successfully deliver PRT to the damaged myocardium, they could decrease *H2Aub* and provide additional protection to the myocardium. The overall synthesis strategy for PRT@PPMP was shown in Fig. 6A. Electron microscopy revealed that PRT@PPMP exhibited a uniform and spherical morphology, with a mean particle size of approximately 25 nm (Fig. 6B, C). The zeta potential was determined to analyze the charge variations and surface modifications of PPM. After loading PPMP with PRT, the zeta potential was altered to -20.54 ± 1.99 mV (Fig. 6D). The internalization of the PRT@PPMP nanospheres within cells is crucial for their therapeutic effectiveness. The PRT@PPMP nanoplatforms functionalized with PCM-SH are expected to demonstrate specificity in their homologous targeting capacity. To demonstrate the specific recognition and targeted delivery of PRT@PPMP to cardiomyocytes (CM), PRT@PPMP was administered to CM and cardiac microvascular endothelial cells (CMECs). The observation of weak PRT@PPMP signals in CMECs indicated the low efficiency of PRT@PPMP uptake. In contrast, the intracellular PRT@PPMP fluorescence signal in the CM was stronger than that in the CMECs (Fig. 6E). Next, the targeting ability of PRT@PPMP in H-R cardiomyocytes was analyzed using confocal laser scanning microscopy (CLSM). Cardiomyocytes incubated with PRT@PPMP exhibited a more intense green fluorescence than PRT@PPM nanospheres (Fig. 6F), suggesting that the PCM-SH-modification facilitated the intracellular uptake of nanocarriers. Moreover, prolonging the co-incubation duration further amplified the intensity of the green fluorescence. After 2 h of incubation, the PRT@PPMP nanosphere-treated group exhibited a higher rate of intracellular uptake than the PRT@PPM nanosphere-treated group. Flow cytometric analysis of the cellular uptake of PRT@PPM and PRT@PPMP by cardiomyocytes revealed that the fluorescence intensity of PRT@PPMP in the cells was significantly higher than that of the PRT@PPM and PBS groups (Fig. 6G). These findings suggested that PCM-SH promoted the intracellular uptake of nanocarriers, thereby exerting a more potent therapeutic effect.

### Therapeutic effects of PRT@PPMP on H-R cardiomyocytes

**Fig. 7:**
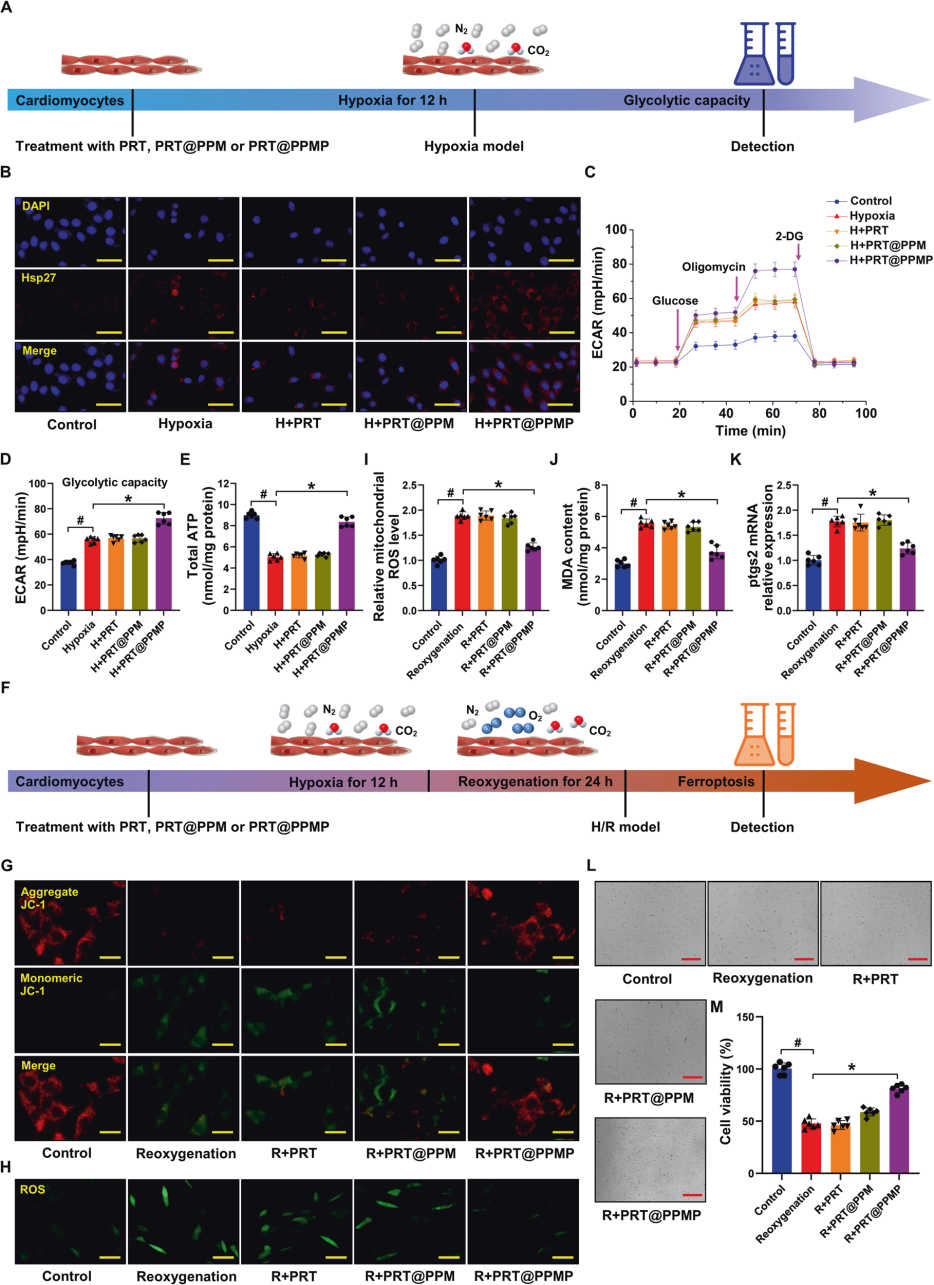
PRT@PPMP reverses myocardial injury by enhancing glycolysis during hypoxia and improving mitochondrial function during reoxygenation. **A** Scheme of the time axis representing the design of hypoxic cell research. **B** Immunofluorescence results showing *Hsp27* expression in control cardiomyocytes and hypoxic cardiomyocytes treated with PRT, PRT@PPM, and PRT@PPMP. **C**, **D** Glycolytic stress analyses of cardiomyocytes using the Seahorse XF analyzer in response to oligomycin, glucose, and 2-DG. **E** Intracellular ATP levels in cardiomyocytes. Data are presented as mean ± SD (n = 6). ^#^*P* < 0.05 vs. Control group; ^*^P < 0.05 vs. Hypoxia group. **F** Scheme of the time axis illustrating the design of H-R-induced cell study. **G** Representative images of JC-1 fluorescence in the control and reoxygenated cardiomyocytes treated with PRT, PRT@PPM, and PRT@PPMP. **H** Immunofluorescence detection of cellular ROS levels in cardiomyocytes. **I** Mitochondrial ROS levels in cardiomyocytes. **J** MDA levels in cardiomyocytes. **K** Relative mRNA levels of *ptgs2* in cardiomyocytes determined using qRT-PCR. **L**–**M** Detection of cardiomyocyte viability. Data are presented as mean ± SD (n = 6). ^#^*P* < 0.05 vs. Control group; ^*^P < 0.05 vs. Reoxygenation group. Scale bars: (**B**, **H**) 50 μm; (**G**) 25 μm; and (**L**) 100 μm.

The effect of PRT@PPMP on cardiomyocyte viability was observed in vitro. At concentrations ≤20 μM, PRT@PPMP displayed minimal effects on cell viability, whereas at a concentration of 40 μM, cell viability was significantly decreased compared to that of untreated cells (Fig. S5A). In addition, investigating the effect of administration time on cell viability revealed no significant alterations in cell viability after 4 days of PRT@PPMP administration at concentrations of 5, 10, and 20 μM (Fig. S5B). PRT effectively suppressed *RING1B* expression in cardiomyocytes, and the inhibitory rate was achieved over 70% at 20 μM (Fig. S5C, E). The time axis scheme in Fig. 7A depicts the glycolytic capacity of cardiomyocytes subjected to 12-h of hypoxia. Cardiomyocytes were pretreated with PBS, PRT, PRT@PPM, or PRT@PPMP (20 μM PRT) before induction of hypoxia. Western blotting and immunofluorescence demonstrated that *Hsp27* levels were significantly decreased in a time-dependent manner (Fig. S6A–C) in vivo. The results of immunofluorescence, western blotting, and qRT-PCR suggested that PRT@PPMP pretreatment effectively stabilized the high expression of *Hsp27* in cardiomyocytes (Fig. S7A, Fig. 7B). Subsequent investigation revealed that PRT@PPMP, but not PRT and PRT@PPM, increased glycolytic flux (Fig. 7C, D) and ATP generation (Fig. 7E) in hypoxic cardiomyocytes.

Figure 7F depicts the time-axis scheme of the design for H-R cardiomyocytes. Cardiomyocytes were pre-treated with PBS, PRT, PRT@PPM, or PRT@PPMP (20 μM PRT). Compared with the control group, the JC-1 monomer exhibited a significant increase in H-R cardiomyocytes. However, this increase was reversed by the PRT@PPMP treatment (Fig. 7G). Moreover, the levels of ROS, MDA, and *ptgs2* in the PRT and PRT@PPM treatment groups showed no obvious changes compared to the reoxygenated group. However, these levels were significantly reduced in the PRT@PPMP group (Fig. 7H–K). The CCK-8 assay results (Fig. 7L–M) revealed that the reoxygenated treatment decreased cell viability, while the PRT@PPMP exhibited increased cell viability, indicating the strong protective effect of PRT@PPMP on cardiomyocytes. These findings suggested that PRT@PPMP promoted glycolysis, improved mitochondrial function, and inhibited ferroptosis in H-R cardiomyocytes by upregulating *Hsp27* expression.

### PRT@PPMP protects the myocardium against ischemia/reperfusion injury

**Fig. 8:**
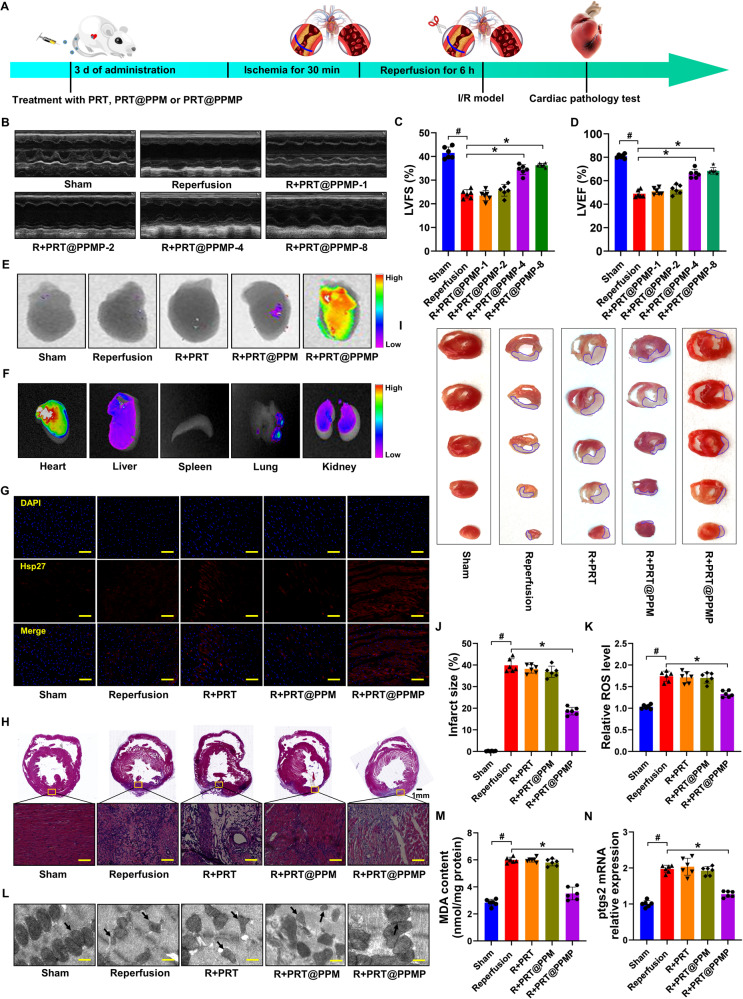
Targeted release of PRT in the myocardium prevents cardiac dysfunction. **A** Scheme of the time axis representing the design of the animal study. **B** Representative echocardiographic images from each group. **C**, **D** Echocardiographic parameters, such as LVEF and LVFS, were calculated in sham and reperfusion rats treated with or without different concentrations of PRT@PPMP. **E** Immunofluorescence study depicting the delivery efficiency of Cy5.5-stained PRT@PPM or PRT@PPMP to the heart 6 h after individualized treatment. **F** Cy5.5-stained PRT@PPMP accumulated in the heart and other organs. **G** Representative images of the heart sections labeled with *Hsp27* fluorescent probe. DAPI (blue, nuclei); *Hsp27* positive (red). **H** Representative morphological analysis using HE staining. **I**–**J** TTC staining revealing the area of myocardial infarction in rats. **K** ROS levels in the cardiac tissue. **L** Electron microscopic analysis of myocardial cells revealing changes in mitochondrial morphology. **M** MDA levels in the cardiac tissue. **N** Relative mRNA levels of *ptgs2* measured in cardiac tissue using qRT-PCR. Data are presented as mean ± SD (n = 6). ^#^*P* < 0.05 vs. Sham group; ^*^P < 0.05 vs. Reperfusion group. Scale bars: (**G**, **H**) 50 μm; (**L**) 2.0 μm.

Figure 8A shows the experimental design of the animal studies. Analysis of cardiac function indicators revealed that the PRT@PPMP-4 and PRT@PPMP-8 (PRT administered at doses of 4 and 8 mg/kg) exhibited superior effectiveness in restoring LVFS and LVEF compared to the reperfusion group. This highlighted the significant treatment efficacy of both PRT@PPMP-4 and PRT@PPMP-8 in enhancing the function of the damaged myocardium (Fig. 8B–D). Therefore, PRT@PPMP-4 (PRT@PPMP) was selected for subsequent experiments. The echocardiographic results confirmed that PRT@PPMP did not affect cardiac function in sham rats (Fig. S8A–C). Next, the distribution of fluorescent signals of the nanocomplex in the heart was quantitatively analyzed using bioimaging techniques. In the PRT@PPM group, the weak fluorescence signals were observed in the heart, whereas in the PRT@PPMP group, the strong fluorescence signals were exhibited, demonstrating good target specificity of PRT@PPMP (Fig. 8E). PCM-SH facilitates the binding of PRT@PPMP to cardiomyocytes, and this interaction is specific to the heart because most of the other tissues or organs, such as the spleen, lungs, liver, and kidneys, exhibited significantly lower distribution of PRT@PPMP (Fig. 8F).

Moreover, immunofluorescence analysis revealed that PRT@PPMP pre-treatment effectively maintained an elevated level of *Hsp27* in the heart (Fig. 8G). Only PRT@PPMP significantly increased the ATP production (Fig. S9A). To investigate the targeted therapeutic effect of PRT@PPMP on MI/RI rats, TTC and HE staining of hearts were performed to detect infarcted areas, and the results demonstrated a significant reduction in infarcted regions with PRT@PPMP (Fig. 8H–J). Ferroptosis markers in MI/RI hearts were examined to investigate the protective effects of PRT@PPMP in vivo. In the model group, exposure to PBS resulted in elevated levels of MDA, ROS, *ptgs2*, and atrophic mitochondria in the myocardial cells, as evidenced by qRT-PCR, ELISA, and electron microscopy. The ferroptosis indicators showed no changes in the PRT and PRT@PPM groups. However, in the PRT@PPMP group, there were significant reduction in ferroptosis indicators, indicating the potent anti-ferroptosis effect of PRT@PPMP on myocardial cells (Fig. 8K–N). Overall, the findings of this study confirmed that PRT@PPMP could specifically target the damaged myocardium and effectively enhance glycolysis while inhibiting ferroptosis, thereby conferring protection to the myocardium against MI/RI.

## Discussion

**Fig. 9:**
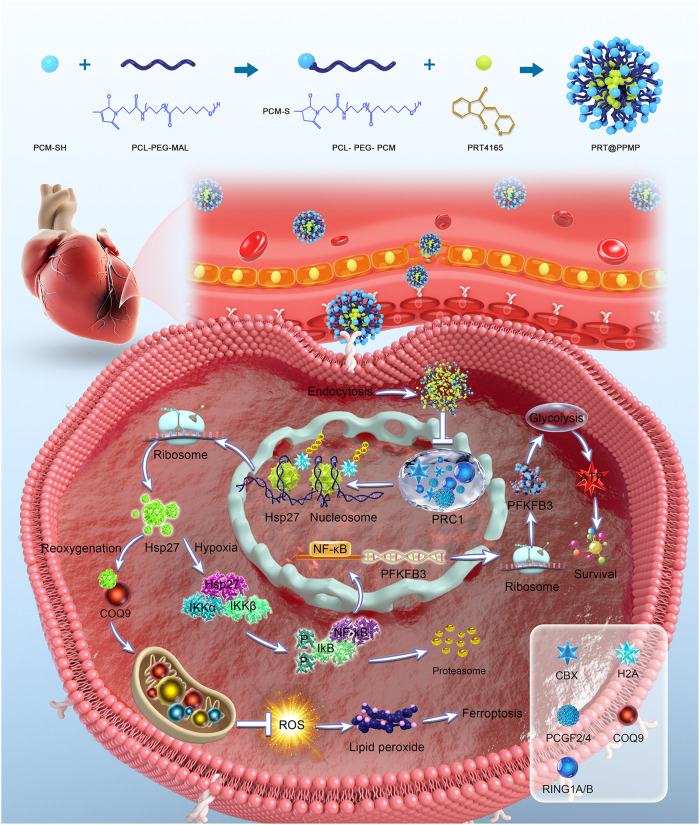
Therapeutic mechanism of PRT@PPMP in MI/RI. PRT@PPMP targets injured cardiomyocytes, leading to the decomposition and release of PRT4165 into the cytoplasm. PRT4165 decreases the expression of *RING1B* and *H2Aub* and increases *Hsp27* expression. *Hsp27* activates the *NF-κB*/*PFKFB3* signaling pathway, leading to increased glycolysis levels during hypoxia. Additionally, *Hsp27* functions as a chaperone of *COQ9* during reoxygenation to improve mitochondrial function and inhibit ferroptosis.

Despite advancements in acute myocardial infarction (AMI) treatment and improved patient survival rates, ischemic heart disease remains a leading cause of mortality worldwide. Therefore, the development of novel cardioprotective strategies is crucial to mitigate the detrimental effects of AMI. Restoring coronary blood flow reperfusion is the most effective approach to improve AMI outcomes [48]. However, reperfusion can also exacerbate myocardial injury [49]. Therefore, identifying effective treatment targets for MI/RI inhibition is of utmost importance. This study reported a significant increase in *Hsp27* levels through histone *H2Aub* in cardiac tissue during ischemia, followed by a decrease during reperfusion. Moreover, the upregulation of *Hsp27* promoted glycolysis under ischemic conditions and stabilized the mitochondrial function during reperfusion. Further mechanistic investigations indicated that *Hsp27* boosted glycolysis by promoting nuclear *NF-κB* translocation, leading to the upregulation of *PFKFB3* expression during ischemia. In addition, *Hsp27* inhibited ferroptosis by improving mitochondrial function through the stabilization of *COQ9*. The present study also introduced a noninvasive, targeted nanocomplex delivery platform using PCM-SH-coated PRT for the treatment of MI/RI. Myocardial cells phagocytized the accumulated PRT@PPMP in the damaged heart, resulting in therapeutic efficacy against MI/RI (Fig. 9). Furthermore, this study explored the intricate mechanism of PRT@PPMP treatment in MI/RI. First, target-specific delivery of PRT@PPMP was successfully accomplished. Upon uptake by cardiomyocytes, PRT@PPMP decomposition triggered the release of PRT into the cytoplasm, ultimately inhibiting *PRC1*. Additionally, reduced *H2Aub* expression stabilized the high *Hsp27* expression, thereby augmenting glycolysis during ischemia and improving mitochondrial function during reoxygenation. This strategy provides a practical approach for implementing PRT treatment for MI/RI.

*HSPs*, a highly conserved protein family activated in response to various physiological and environmental challenges such as hyperoxia and hypoxia, as well as emotional and mechanical stress, play a critical role in maintaining homeostasis of cells under stress or allowing cell survival in lethal environments [50–52]. Myocardial cells, with their high metabolic demands and abundant signal transduction pathways, are particularly reliant on chaperones for survival. *Hsp27*, a prominent member of the small *HSP* family, is highly expressed in the myocardium and exhibits cardioprotective properties [53]. Small *HSPs* regulate protein folding, provide significant protection against apoptosis and oxidative stress, and contribute to the maintenance of sarcomeric structure. Despite the well-established role of *Hsp27* in the stress response and characterization, there is currently no practical approach for maintaining its activity in MI/RI for cardiac protection. In this study, *Hsp27* was identified as a glycolysis-associated gene, and maintaining a high level of *Hsp27* enhanced glycolysis via the *NF-κB*/*PFKFB3* signaling pathway. Studies have confirmed that *Hsp27* exhibits potent chaperone activity against amyloid and amorphous aggregation of proteins, such as *Tau, α-synuclein*, and *SOD1* [54, 55]. Researchers have revealed that small heat shock proteins, including *Hsp27*, function as chaperone systems within the mitochondrial intermembrane space [56]. Similarly, the present study indicated that *Hsp27* also enhanced mitochondrial function by stabilizing *COQ9* protein expression rather than through changes in mRNA levels. Therefore, *Hsp27* acted as a molecular chaperone, facilitated the proper protein folding of *COQ9*. These findings indicated that *Hsp27* played a protective role in the myocardium by promoting glycolysis and improving mitochondrial function.

Published literature has identified covalent modification of histone core models in multiple DNA-based processes, including transcription, DNA repair, and replication [57]. Histone ubiquitination is implicated in various cellular processes. In particular, *H2Aub*, which primarily occurs at *Lys119*, is crucial because of its association with histones and its extensive range of modifications [58]. *H2Aub* is primarily correlated with gene repression in chromatin and is strongly associated with *PRC1*, the primary ligase enzyme for *H2Aub* [59]. Recent studies have also shown that *H2Aub* is linked to the emergence of multiple diseases. For example, *BAP1* has been found to reduce *H2Aub* occupancy of the *SLC7A11* promoter, leading to the suppression of *SLC7A11* expression and contributing to elevated ferroptosis and lipid peroxidation [60]. Additionally, *TRIM37* orchestrates the progression of renal cell carcinoma by regulating histone *H2Aub* [61]. However, the mechanisms underlying *PRC1*-regulated *H2Aub* suppression of target genes are not well understood in the context of cardiovascular disease. In this study, ChIP-seq analysis revealed that *H2Aub* regulated 1289 genes in the MI model. Moreover, KEGG analysis showed that *H2Aub*-regulated genes were enriched in the *MAPK* signaling pathway, which included *Hsp27*. The experimental results indicated reduced levels of *H2Aub* and *RING1B* in the hearts of MI rats and hypoxic cardiomyocytes. Enforcing *RING1B* expression in hypoxic cardiomyocytes increased *H2Aub* protein expression while simultaneously reducing the mRNA and protein levels of *Hsp27*. Furthermore, ChIP-qPCR results confirmed that *H2Aub* combined with *Hsp27*. These data suggested that *H2Aub* directly inhibited *Hsp27* expression.

Recently, researchers have shifted their attention toward targeted therapies for MI/RI. This approach involves the use of a peptide-conjugated nanoparticle (PRT@PPMP) for the targeted delivery of a PPM-conjugated 21-mer peptide, namely PCM-SH, to the heart. In vitro and in vivo studies have demonstrated the preferential binding of PRT@PPMP to cardiomyocytes compared to the scrambled control, with the PRT@PPMP signal being prominently distributed in the rat heart. This indicated that PCM-SH could efficiently guide nanoparticles toward the heart. PRT accumulated in the injured heart was phagocytosed by cardiomyocytes, resulting in long-term treatment efficacy against MI/RI. Furthermore, the specific mechanism by which PRT@PPMP treatment affected MI/RI was investigated, revealing enhanced glycolysis, improved mitochondrial function, and reduced ferroptosis in cardiomyocytes. Therefore, this study provides a novel strategy for the clinical treatment of MI/RI.

In summary, this study reveals the involvement of *H2Aub* in the development of MI/RI. A novel regulatory pathway in glycolysis involving the *H2Aub*/*Hsp27*/*NF-κB*/*PFKFB3* axis was established through mechanistic studies. Furthermore, the upregulation of *Hsp27* expression improved mitochondrial function through its role as a chaperone for *COQ9*. More importantly, a noninvasive, targeted nanocomplex delivery platform was developed using PPMP-coated PRT to treat MI/RI. These findings not only expand our understanding of the potential therapeutic role of PRT in cardiovascular disease, but also highlight the importance of developing therapeutic strategies that target the *H2Aub* pathway.

## Materials and methods

### Micelle preparation and characterization

PCL-PEG-MAL or PCL-PEG-PCM (25 mg) was dissolved in 5 ml methanol, mixed with 15 mg of PRT, and subsequently dissolved in dichloromethane (5 ml). The thin solid film resulting from the evaporation of the solvent was dissolved in 10 ml PBS solution, gently agitated for 1 h, and then filtered using a 0.2 μm syringe filter to remove non-encapsulated or precipitated PRT. PRT-free blank micelles were also prepared.

### Establishment of the MI/RI model

MI/RI was performed as previously described [62]. Briefly, male rats were anesthetized through isoflurane gas inhalation and placed in the supine position, where they were surgically assisted with breathing using an animal ventilator. Left thoracotomy was performed through a horizontal incision in the fourth intercostal space, and the left anterior descending coronary artery was ligated around the PE-10 tube with 6-0 sutures. Occlusion of the left anterior descending coronary artery was confirmed by elevation of the ST segment on electrocardiogram. After 30 min, the PE-10 tube was removed and the reperfusion was performed for 6 h. The hearts were harvested for subsequent experiments. The rats were randomly divided into five groups with six rats in each group, as follows: (1) sham group rats underwent a sham operation and received vehicle (PBS, caudal vein injection); (2) reperfusion group rats were subjected to MI/RI and received vehicle (PBS, caudal vein injection); and (3–5) PRT groups rats were received PRT, PRT@PPM, or PRT@PPMP (4 mg/kg, caudal vein injection). PRT, PRT@PPM, or PRT@PPMP was administered once daily for three days, and the ischemia-reperfusion injury model was established immediately after the last administration. In this study, the rats were subjected to MI/RI and treated with the above interventions randomly. Efficacy was evaluated based on morphology, transthoracic echocardiography, and biomarker analyses in a blinded fashion.

### Echocardiographic evaluation

Echocardiography was performed following a previously described method [63]. The rats were anesthetized using isoflurane gas inhalation, and their chest hair was removed using a depilation agent. The chest was coated with ultrasound gel and scanned using a contact scanning head.

### TTC and HE staining

The rats were euthanized after ischemia or reperfusion and their hearts were obtained. The hearts were subsequently stored at –20 °C for 25 min. The heart tissue was sectioned into five pieces and placed in 2% TTC at 37 °C for 15 min in the dark [64]. The infarct size was determined using ImageJ software. Moreover, several heart sections (4–5 μm thick) were prepared and stained with HE for histopathological analysis [65].

### Electron microscopy

Mitochondrial alterations in cardiomyocytes during MI/RI were observed using electron microscopy as described previously [66].

### Detection of reactive oxygen species (ROS), lipid peroxide, *NF-κB* content, and *IKK* activity

ROS, lipid peroxide, *NF-κB* content, and *IKK* activity were measured according to the manufacturer’s instructions.

### Quantitative reverse-transcription PCR (qRT-PCR)

Total RNA from cardiomyocytes and cardiac tissues was isolated and reverse transcribed. The mRNA levels were detected using qRT-PCR and normalized to β-actin expression levels. Primer sequences used in this study are listed in Table S1.

### Western blot

Myocardial tissue or cultured cardiomyocytes were lysed using RIPA lysis buffer for western blot analysis. Protein extracts (50 μg) were isolated using sodium dodecyl sulfate-polyacrylamide gel electrophoresis (SDS-PAGE), transferred to a nitrocellulose membrane, and probed with primary antibodies overnight at 4 °C (Table S2**)**. Enhanced chemiluminescence (ECL) reagents were used to visualize the bands using an Imaging System (LI-COR Biosciences).

### Immunofluorescence assay

Immunofluorescence assays were performed using antibodies against *H2AX*, *Hsp27*, and *RING1B*. The samples were incubated overnight at 4 °C with the primary antibodies. Next, the samples were rinsed three times with PBS and incubated with species-specific secondary antibodies.

The different uptake capacities of PRT@PPMP and PRT@PPM by cardiomyocytes were validated using immunofluorescence. PRT@PPM and PRT@PPMP were labeled with green dye.

### Primary cardiomyocyte isolation

Myocardial tissue was isolated from 1–2-day-old rats. Cardiomyocyte isolation involved digestion of heart tissue with 2.5 mg/ml trypsin (Aladdin Co., Ltd., Shanghai, China) and perfusion with digestion buffer containing 2 mg/ml DNase I (Sigma, D7291). Cardiomyocytes were collected from the tissue piece suspension by centrifugation and cultured in DMEM (HyClone, C11995500BT) supplemented with 20% FBS and 40 IU/ml penicillin (Sigma, P3032).

### Preparation of H-R cardiomyocyte model

Cardiomyocytes were subjected to a 12-h hypoxia treatment, followed by a 24-h reoxygenation to replicate MI/RI in vitro. Cells were cultured in a hypoxic incubator (5% CO_2_, 95% N_2_) at 37 °C. Following the standard culture method, cells were reoxygenated. Cardiomyocytes were randomly classified into five groups: (1) control group cells were incubated under normal conditions; (2) reoxygenated group cells were exposed to hypoxia for 12 h, followed by reoxygenation for 24 h; and (3–5) PRT group cells were treated with PRT, PRT@PPM, or PRT@PPMP, and then subjected to the same hypoxia and reoxygenation protocol.

### Metabolite assays

Lactate and ATP levels were measured using the lactate production assay kit (Solarbio) and the ATP assay kit (Solarbio), respectively, following the manufacturer’s protocol.

### Analysis of the extracellular acidification rate (ECAR)

The cells were inoculated into XF24 microporous cell culture plates. The culture medium was replaced following adherence of the cells, and the cells were washed and incubated for 60 min at 37 °C in a CO_2_-free incubator. Next, glucose, oligomycin, and 2-deoxyglucose were sequentially added. Measurement and software analyses were conducted using the XF glycolysis stress test report generator following the manufacturer’s protocol.

### Cell viability assay

Cardiomyocytes were cultured and seeded into 96-well plates. Cell viability was assessed using the Cell counting kit-8 (CCK-8; Dojindo Molecular Technologies), following the manufacturer’s guidelines.

### siRNA construction and transfection

Small interfering RNA (siRNA) sequences were synthesized by Gene Pharma (Shanghai Gene Pharma, China). The primer sequences used were listed in Table S3. Cardiomyocytes were incubated in 5 ml serum-free medium for 4–6 h. siRNA and X-treme GENE siRNA (Invitrogen, Carlsbad, CA, USA) were mixed separately with 300 μl serum-free medium. The two mixtures were combined and incubated at room temperature. Finally, the mixture was added to the cardiomyocytes and incubated at 37 °C for 24 h.

### Luciferase reporter assays

The *PFKFB3* 3’-UTR full-length sequence was amplified. Cells were seeded in triplicate in 48-well plates. Each plate was co-transfected with 40 ng/well luciferase reporter vector and 10 pmol *NF-κB* mimic or mimic control using Lipofectamine 2000 (Invitrogen). Cells were lysed 24 h after transfection, and luciferase activity was determined.

### Co-immunoprecipitation

Immunoprecipitation assay was performed according to the protocol described in a previous study [7]. The treated samples were incubated with control IgG or antibodies overnight at 4 °C, followed by a 4-h incubation with 50 μl protein G Sepharose. The samples were denatured, loaded onto an SDS-PAGE gel, and transferred to a nitrocellulose membrane. The membranes were then incubated with primary antibodies, followed by secondary antibodies.

### Statistical analysis

Data analysis was performed using Statistical Product and Service Solutions (SPSS) 19.0 statistical software (SPSS Inc., Chicago, IL, USA) and Prism 9.0. Quantitative data are expressed as mean ± SD. Shapiro–Wilks test was used to explore whether the data were normally distributed. Brown-Forsythe test was used for group variances analysis. One-way analysis of variance (ANOVA) was used to analyze multiple group comparisons, and *P* < 0.05 was considered statistically significant.

### Supplementary information

Supplemental Information

Original western blots

## Data Availability

The RNA-seq and ChIP-seq data have been uploaded to the NCBI database (GEO: GSE225371). Additional data required from this study are available from the corresponding author upon reasonable request.

## References

[1] Benjamin EJ, Muntner P, Alonso A, Bittencourt MS, Callaway CW, Carson AP (2019). Heart Disease and Stroke Statistics-2019 Update: A Report From the American Heart Association. Circulation.

[2] Rookyard AW, Paulech J, Thyssen S, Liddy KA, Puckeridge M, Li DK (2021). Profile of reversible and irreversible cysteine redox post-translational modifications during myocardial ischemia/reperfusion injury and antioxidant intervention. Antioxid Redox Signaling.

[3] Song R, Dasgupta C, Mulder C, Zhang L (2022). MicroRNA-210 controls mitochondrial metabolism and protects heart function in myocardial infarction. Circulation.

[4] Li Y, Xiong Z, Yan W, Gao E, Cheng H, Wu G (2020). Branched chain amino acids exacerbate myocardial ischemia/reperfusion vulnerability via enhancing GCN2/ATF6/PPAR-α pathway-dependent fatty acid oxidation. Theranostics.

[5] DeBerardinis RJ, Lum JJ, Hatzivassiliou G, Thompson CB (2008). The biology of cancer: metabolic reprogramming fuels cell growth and proliferation. Cell Metab.

[6] Tian R, Abel ED (2001). Responses of GLUT4-deficient hearts to ischemia underscore the importance of glycolysis. Circulation.

[7] Zheng Y, Wan G, Yang B, Gu X, Lin J Cardioprotective natural compound pinocembrin attenuates acute ischemic myocardial injury via enhancing glycolysis. Oxid Med Cell Longev. 2020;2020:4850328. 10.1155/2020/4850328 PMC7644300 33178386

[8] Beltran C, Pardo R, Bou-Teen D, Ruiz-Meana M, Villena JA, Ferreira-González I (2020). Enhancing glycolysis protects against myocardial ischemia-reperfusion injury by reducing ROS production. Metabolites.

[9] Zhang B, Jiang H, Wu J, Cai Yun, Dong Z, Zhao Y (2021). m6A demethylase FTO attenuates cardiac dysfunction by regulating glucose uptake and glycolysis in mice with pressure overload-induced heart failure. Signal Transduct Target Ther.

[10] Hartl FU, Bracher A, Hayer-Hartl M (2011). Molecular chaperones in protein folding and proteostasis. Nature.

[11] Tyedmers J, Mogk A, Bukau B (2010). Cellular strategies for controlling protein aggregation. Nat Rev Mol Cell Biol.

[12] Liu Z, Zhang S, Gu J, Tong Y, Li Y, Gui X (2020). Hsp27 chaperones FUS phase separation under the modulation of stress-induced phosphorylation. Nat Struct Mol Biol.

[13] Noddings CM, Wang RY, Johnson JL, Agard DA (2022). Structure of Hsp90-p23-GR reveals the Hsp90 client-remodelling mechanism. Nature.

[14] Choi S, Chen M, Cryns VL, Anderson RA (2019). A nuclear phosphoinositide kinase complex regulates p53. Nat Cell Biol.

[15] Haslbeck M, Franzmann T, Weinfurtner D, Buchner J (2005). Some like it hot: the structure and function of small heat-shock proteins. Nat Struct Mol Biol.

[16] Almela P, Cuenca-Bermejo L, Yuste JE, Estrada C, Pablos VD, Bautista-Hernández V (2020). Cardiac noradrenaline turnover and heat shock protein 27 phosphorylation in dyskinetic monkeys. Mov Disord.

[17] Lu XY, Chen L, Cai X, Yang H (2008). Overexpression of heat shock protein 27 protects against ischaemia/reperfusion-induced cardiac dysfunction. Cardiovasc Res.

[18] Kornberg RD, Lorch Y (1999). Twenty-five years of the nucleosome, fundamental particle of the eukaryote chromosome. Cell.

[19] Zentner GE, Henikoff S (2013). Regulation of nucleosome dynamics by histone modifications. Nat Struct Mol Biol.

[20] Tessarz P, Kouzarides T (2014). Histone core modifications regulating nucleosome structure and dynamics. Nat Rev Mol Cell Biol.

[21] Mattiroli F, Vissers JH, van Dijk WJ, Ikpa P, Citterio E, Vermeulen W (2012). RNF168 ubiquitinates K13-15 on H2A/H2AX to drive DNA damage signaling. Cell.

[22] Kalb R, Mallery DL, Larkin C, Huang JT, Hiom K (2014). BRCA1 is a histone-H2A-specific ubiquitin ligase. Cell Rep.

[23] Zhou W, Wang X, Rosenfeld MG (2009). Histone H2A ubiquitination in transcriptional regulation and DNA damage repair. Int J Biochem Cell Biol.

[24] Higashi M, Inoue S, Ito T (2010). Core histone H2A ubiquitylation and transcriptional regulation. Exp Cell Res.

[25] Hengbin W, Liangjun W, Hediye E, Miguel V, Paul T, Richard SJ (2004). Role of histone H2A ubiquitination in Polycomb silencing. Nature.

[26] Ben-Saadon R, Zaaroor D, Ziv T, Ciechanover A (2006). The polycomb protein Ring1B generates self atypical mixed ubiquitin chains required for its in vitro histone H2A ligase activity. Mol Cell.

[27] Zaaroor-Regev D, de Bie P, Scheffner M, Noy T, Shemer R, Heled M (2010). Regulation of the polycomb protein Ring1B by self-ubiquitination or by E6-AP may have implications to the pathogenesis of Angelman syndrome. Proc Natl Acad Sci USA.

[28] Chen H, Zhou J, Chen H, Liang J, Xie C, Gu X (2022). Bmi-1-RING1B prevents GATA4-dependent senescence-associated pathological cardiac hypertrophy by promoting autophagic degradation of GATA4. Clin Transl Med.

[29] Blin G, Liand M, Mauduit C, Chehade H, Benahmed M, Simeoni U (2020). Maternal exposure to high-fat diet induces long-term derepressive chromatin marks in the heart. Nutrients.

[30] McGuire MJ, Samli KN, Johnston SA, Brown KC (2004). In vitro selection of a peptide with high selectivity for cardiomyocytes in vivo. J Mol Biol.

[31] Wei X, Zhang Y, Li C, Ai K, Li K, Li H (2020). The evolutionarily conserved MAPK/Erk signaling promotes ancestral T-cell immunity in fish via c-Myc-mediated glycolysis. J Biol Chem.

[32] Wang F, Qi XM, Wertz R, Mortensen M, Hagen C, Evans J (2020). 6p38γ MAPK is essential for aerobic glycolysis and pancreatic tumorigenesis. Cancer Res.

[33] Zhang Y, Liu W, Zhong Y, Li Q, Wu M, Yang L, et al. Metformin Corrects Glucose Metabolism Reprogramming and NLRP3 Inflammasome-Induced Pyroptosis via Inhibiting the TLR4/NF-κB/PFKFB3 Signaling in Trophoblasts: Implication for a Potential Therapy of Preeclampsia. Oxid Med Cell Longev. 2021;2021:1806344. 10.1155/2021/1806344 PMC8601820 34804360

[34] Ren X, Chen C, Luo Y, Liu M, Li Y, Zheng S (2020). lncRNA-PLACT1 sustains activation of NF-κB pathway through a positive feedback loop with IκBα/E2F1 axis in pancreatic cancer. Mol Cancer.

[35] Ji J, Ding K, Luo T, Zhang X, Chen A, Zhang D (2021). TRIM22 activates NF-κB signaling in glioblastoma by accelerating the degradation of IκBα. Cell Death Differ.

[36] Horváth I, Multhoff G, Sonnleitner A, Vígh L (2008). Membrane associated stress proteins: More than simply chaperones. Biochem Biophys Acta.

[37] Bolli R (1992). Myocardial ‘stunning’ in man. Circulation.

[38] Lochner A, Marais E, Genade S, Huisamen B, Du Toit EF, Moolman JA (2009). Protection of the ischaemic heart: Investigations into the phenomenon of ischaemic preconditioning. Cardiovasc.

[39] Hao X, Zhang S, Timakov B, Zhang P (2007). The HSP-27 gene is not required for Drosophila development but its activity is associated with starvation resistance. Cell Stress Chaperones.

[40] Zhang HL, Jia KY, Sun D, Yang M (2019). Protective effect of HSP27 in atherosclerosis and coronary heart disease by inhibiting reactive oxygen species. J Cell Biochem.

[41] Wyttenbach A, Sauvageot O, Carmichael J, Diaz-Latoud C, Arrigo AP, Rubinsztein DC (2002). Heat shock protein 27 prevents cellular polyglutamine toxicity and suppresses the increase of reactive oxygen species caused by huntingtin. Hum Mol Genet.

[42] Abdel-Rahman EA, Hosseiny S, Aaliya A, Adel M, Yasseen B, Al-Okda A (2021). Sleep/wake calcium dynamics, respiratory function, and ROS production in cardiac mitochondria. J Adv Res.

[43] Nolfi-Donegan D, Braganza A, Shiva S (2020). Mitochondrial electron transport chain: oxidative phosphorylation, oxidant production, and methods of measurement. Redox Biol.

[44] Lopez-Fabuel Irene, Le Douce Juliette, Logan Angela, James AndrewM, Bonvento Gilles, Murphy MichaelP (2016). Complex I assembly into supercomplexes determines differential mitochondrial ROS production in neurons and astrocytes. Proc Natl Acad Sci USA.

[45] Adriaenssens Elias, Asselbergh Bob, Rivera-Mejías Pablo, Bervoets Sven, Vendredy Leen, De Winter Vicky (2023). Small heat shock proteins operate as molecular chaperones in the mitochondrial intermembrane space. Nat Cell Biol.

[46] Cao R, Tsukada Y, Zhang Y (2005). Role of Bmi-1 and Ring1A in H2A ubiquitylation and Hox gene silencing. Mol Cell.

[47] Mailand N, Bekker-Jensen S, Faustrup H, Melander F, Bartek J, Lukas C (2007). RNF8 ubiquitylates histones at DNA double-strand breaks and promotes assembly of repair proteins. Cell.

[48] Ortiz Galeano I, Fariña-López RM, Insaurralde Rodríguez SA, Chirico Achinelli, CE (2019). High blood pressure and other cardiovascular risk factors in students of the national university of Asunción-Paraguay. Rev Fac Cien Med Univ Nac Cordoba.

[49] Jiang Wen, Zhang Yuxiang, Zhang Wei, Pan Xiaomei, Liu Jieyu, Chen Qiang (2023). Hirsutine ameliorates myocardial ischemia-reperfusion injury through improving mitochondrial function via CaMKII pathway. Clin Exp Hypertens.

[50] Young JC, Agashe VR, Siegers K, Hartl FU (2004). Pathways of chaperone-mediated protein folding in the cytosol. Nat Rev Mol Cell Biol.

[51] Khalil AA, Kabapy NF, Deraz SF, Smith C (2011). Heat shock proteins in oncology: diagnostic biomarkers or therapeutic targets?. Biochim Biophys Acta.

[52] Lebret T, Watson RW, Fitzpatrick JM (2003). Heat shock proteins: their role in urological tumours. J Urol..

[53] Mymrikov EV, Seit-Nebi AS, Gusev NB (2011). Large potentials of small heat shock proteins. Physiol Rev.

[54] Freilich R, Betegon M, Tse E, Mok SA, Julien O, Agard DA (2018). Competing protein-protein interactions regulate binding of Hsp27 to its client protein tau. Nat Commun.

[55] Cox D, Whiten DR, Brown JW, Horrocks MH, Gil RS, Dobson CM (2018). The small heat shock protein Hsp27 binds alpha-synuclein fibrils, preventing elongation and cytotoxicity. J Biol Chem.

[56] Baughman HER, Pham TT, Adams CS, Nath A, Klevit RE (2020). Release of a disordered domain enhances HspB1 chaperone activity toward tau. Proc Natl Acad Sci USA.

[57] Zhang Y (2003). Transcriptional regulation by histone ubiquitination and deubiquitination. Genes Dev.

[58] Fursova NA, Blackledge NP, Nakayama M, Ito S, Koseki Y, Farcas AM (2019). Synergy between variant PRC1 complexes defines polycomb-mediated gene repression. Mol Cell.

[59] Scelfo A, Fern´andez-P´erez D, Tamburri S, Zanotti M, Lavarone E, Soldi M (2019). Functional landscape of PCGF proteins reveals both RING1A/B-Dependent-and RING1A/B-Independent-Specific activities. Mol Cell.

[60] Zhang Y, Shi J, Liu X, Feng L, Gong Z, Koppula P (2018). BAP1 links metabolic regulation of ferroptosis to tumour suppression. Nat Cell Biol.

[61] Miao C, Liang C, Li P, Liu B, Qin C, Yuan H (2021). TRIM37 orchestrates renal cell carcinoma progression via histone H2A ubiquitination dependent manner. J Exp Clin Cancer Res.

[62] Sawashita Y, Hirata N, Yoshikawa Y, Terada H, Tokinaga Y, Yamakage M (2020). Remote ischemic preconditioning reduces myocardial ischemia-reperfusion injury through unacylated ghrelin-induced activation of the JAK/STAT pathway. Basic Res Cardiol.

[63] Chakouri N, Farah C, Matecki S, Amedro P, Vincenti M, Saumet L (2020). Screening for in-vivo regional contractile defaults to predict the delayed Doxorubicin Cardiotoxicity in Juvenile Rat. Theranostics.

[64] Zhou J, Liu W, Zhao X, Xian Y, Wu W, Zhang X (2021). Natural melanin/alginate hydrogels achieve cardiac repair through ROS scavenging and macrophage polarization. Adv Sci (Weinh).

[65] Wang R, Wang M, Zhou J, Dai Z, Sun G, Sun X (2020). Calenduloside E suppresses calcium overload by promoting the interaction between L-type calcium channels and Bcl2-associated athanogene 3 to alleviate myocardial ischemia/reperfusion injury. J Adv Res.

[66] Zhou H, Li D, Zhu P, Ma Q, Toan S, Wang J (2018). Inhibitory effect of melatonin on necroptosis via repressing the Ripk3-PGAM5-CypD-mPTP pathway attenuates cardiac microvascular ischemia-reperfusion injury. J Pineal Res.

