# Supplementary material for: Upregulation of Hsp27 via further inhibition of histone H2A ubiquitination confers protection against myocardial ischemia/reperfusion injury by promoting glycolysis and enhancing mitochondrial function
Source: Cell Death Discov. 2023 Dec 19;9:466. doi: 10.1038/s41420-023-01762-x (PMC10730859; doi:10.1038/s41420-023-01762-x)
Supplement: Supplementary file 1 — Supplemental Information [file 41420_2023_1762_MOESM1_ESM.docx]

**Supplemental Information**


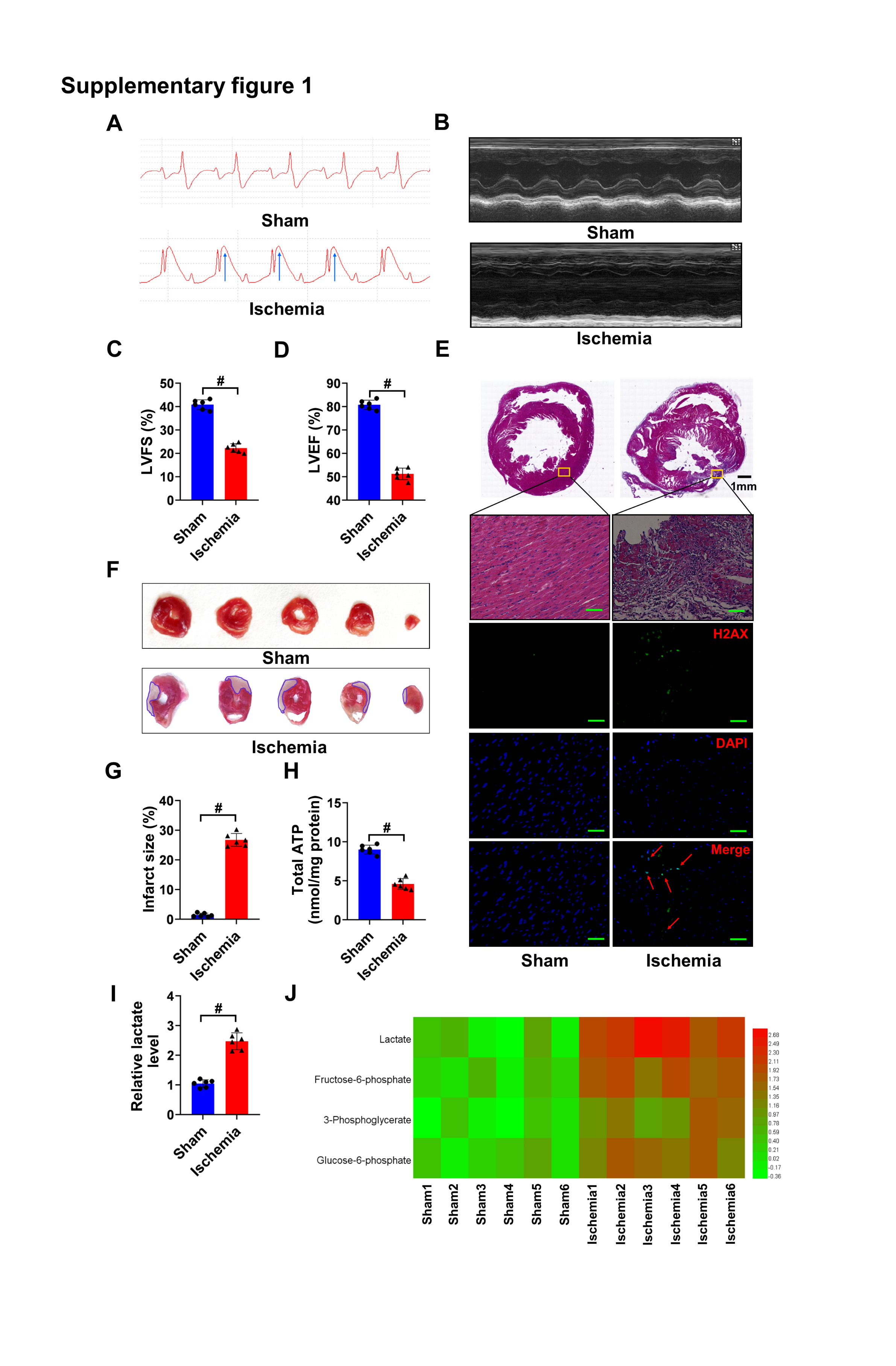


**Supplementary figure S1. Glycolysis is induced in ischemia hearts**

(A) The electrocardiogram of rats induced by ischemia for 30 min. (B-D) Representative images and statistical graphs for echocardiographic parameters of ischemia hearts in rats. The echocardiographic parameters including left ventricular fractional shortening (LVFS) and left ventricular ejection fraction (LVEF) were measured. (E) Representative sections of heart stained for HE staining and *H2AX* immunofluorescence staining in rats induced by ischemia for 30 min. (F-G) TTC staining detected the area of myocardial infarction in rats. (H) ATP levels were detected in cardiac tissue. (I) Lactate levels were detected in cardiac tissue. (J) Heat map of glycolytic intermediates 30 min after ischemia stimulation. Ischemia group: Hearts of rats were exposed to ischemia for 30 min. Data were represented by mean ± SD (n=6). ^#^*P* < 0.05 vs. Sham group. Scale bars, 50 μm.


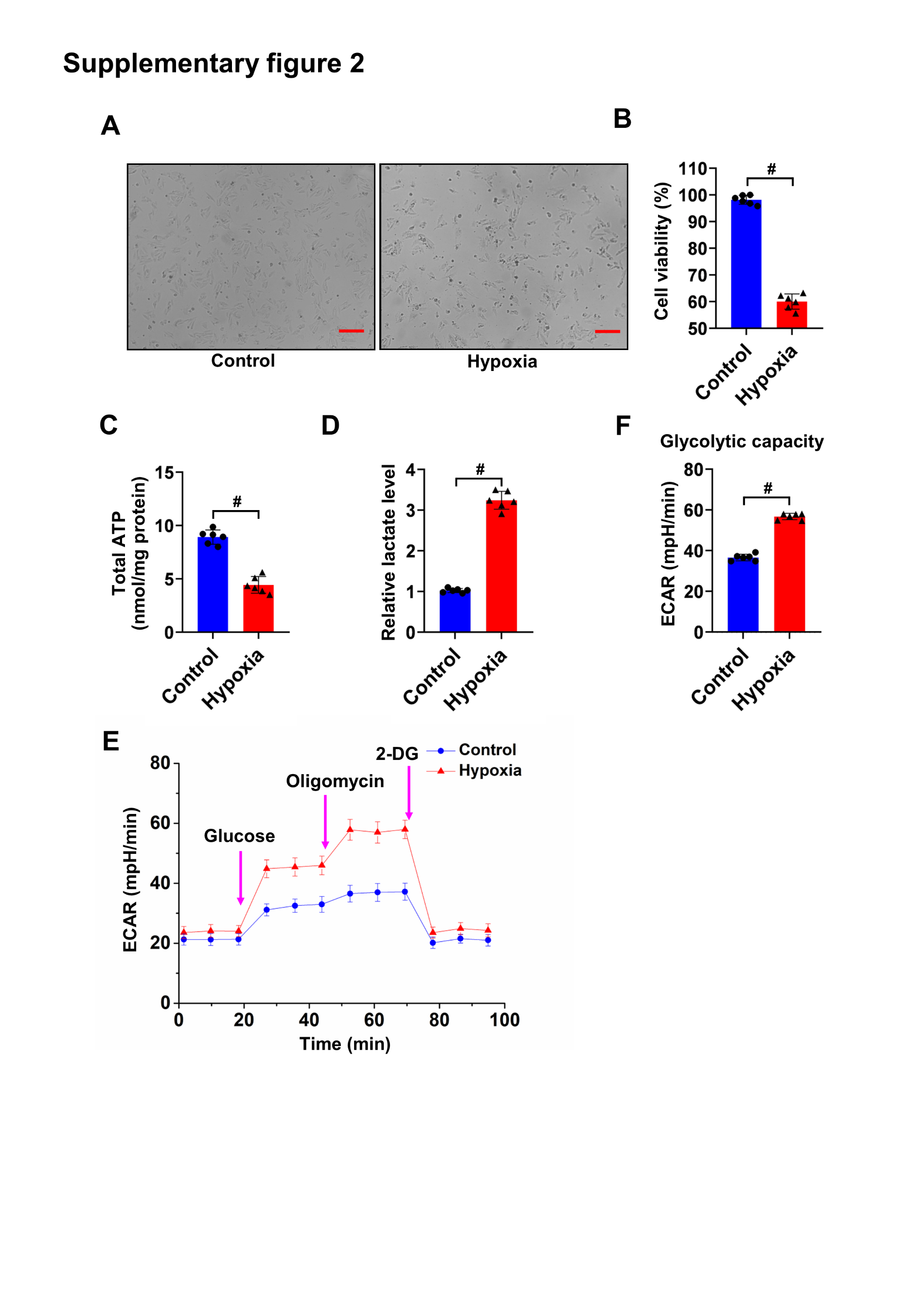


**Supplementary figure S2. Hypoxia promotes cardiomyocytes glycolysis**

(A-B) Cell viability of cardiomyocytes induced by hypoxia for 12 h was detected. (C) Intracellular ATP levels were detected in cardiomyocytes. (D) Lactate levels were detected in cardiomyocytes. (E-F) Seahorse XF assay comparing ECAR in hypoxia for 12 h cardiomyocytes with control cells. Hypoxia group: cardiomyocytes were exposed to hypoxia for 12 h. Data were represented by mean ± SD (n=6). ^#^*P* < 0.05 vs. Control group. Scale bars, 100 μm.


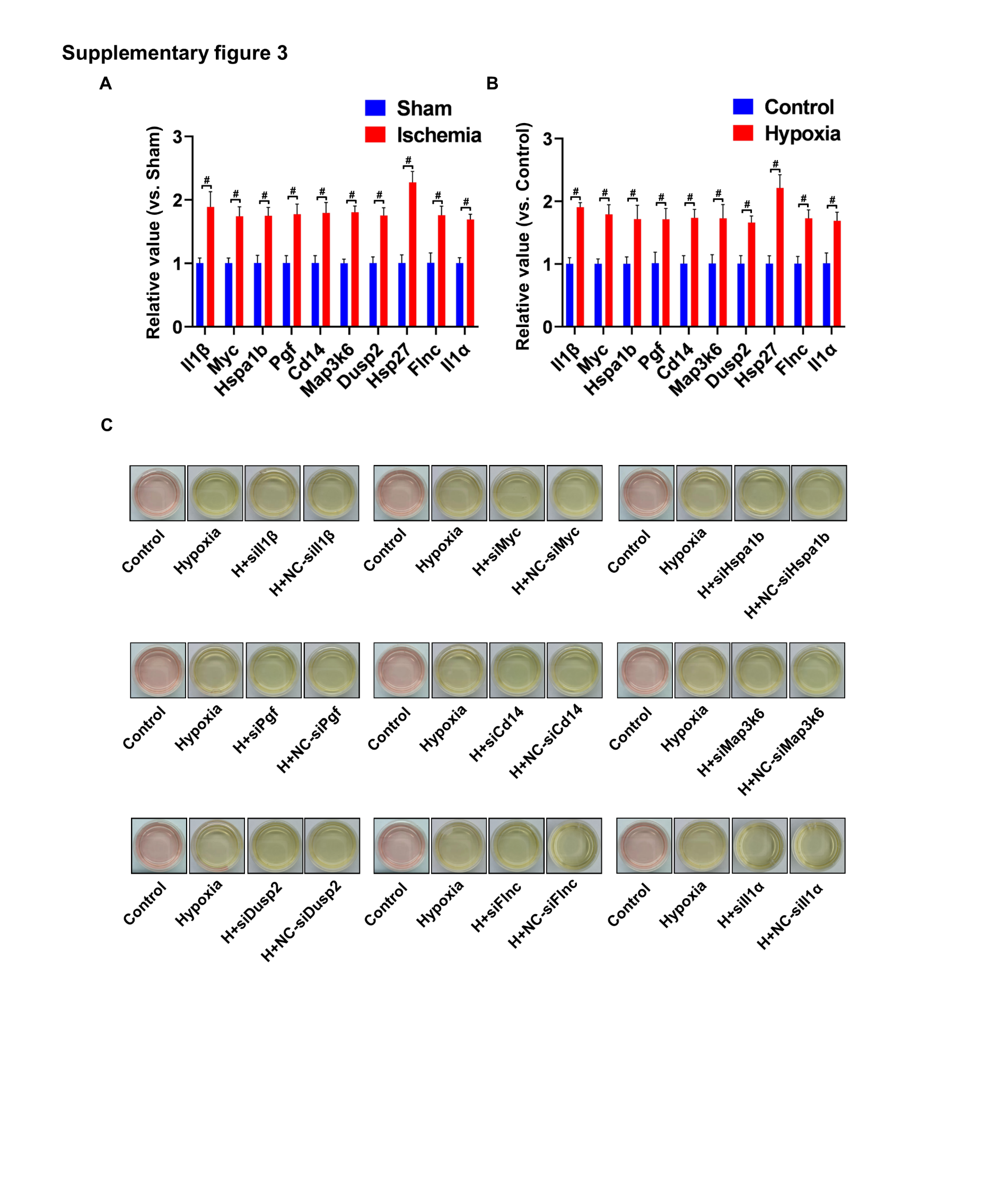


**Supplementary figure S3.** **Culture medium acidification after depletion of genes in *MAPK* signal pathway in cardiomyocytes**

(A) Relative mRNA level of genes in *MAPK* signal pathway were determined by qRT-PCR in cardiac tissue of rats treated by ischemia for 30 min. Ischemia group: Hearts of rats were exposed to ischemia for 30 min. Data were represented by mean ± SD (n=6). ^#^ *P*< 0.05 vs. Sham group. (B) Relative mRNA level of genes in *MAPK* signal pathway were determined by qRT-PCR in cardiomyocytes induced by hypoxia for 12 h. Data were represented by mean ± SD (n=6). ^#^*P* < 0.05 vs. Control group. (C) Culture medium acidification after knockdown of genes in *MAPK* signal pathway in cardiomyocytes. Hypoxia group: cardiomyocytes were exposed to hypoxia for 12 h.


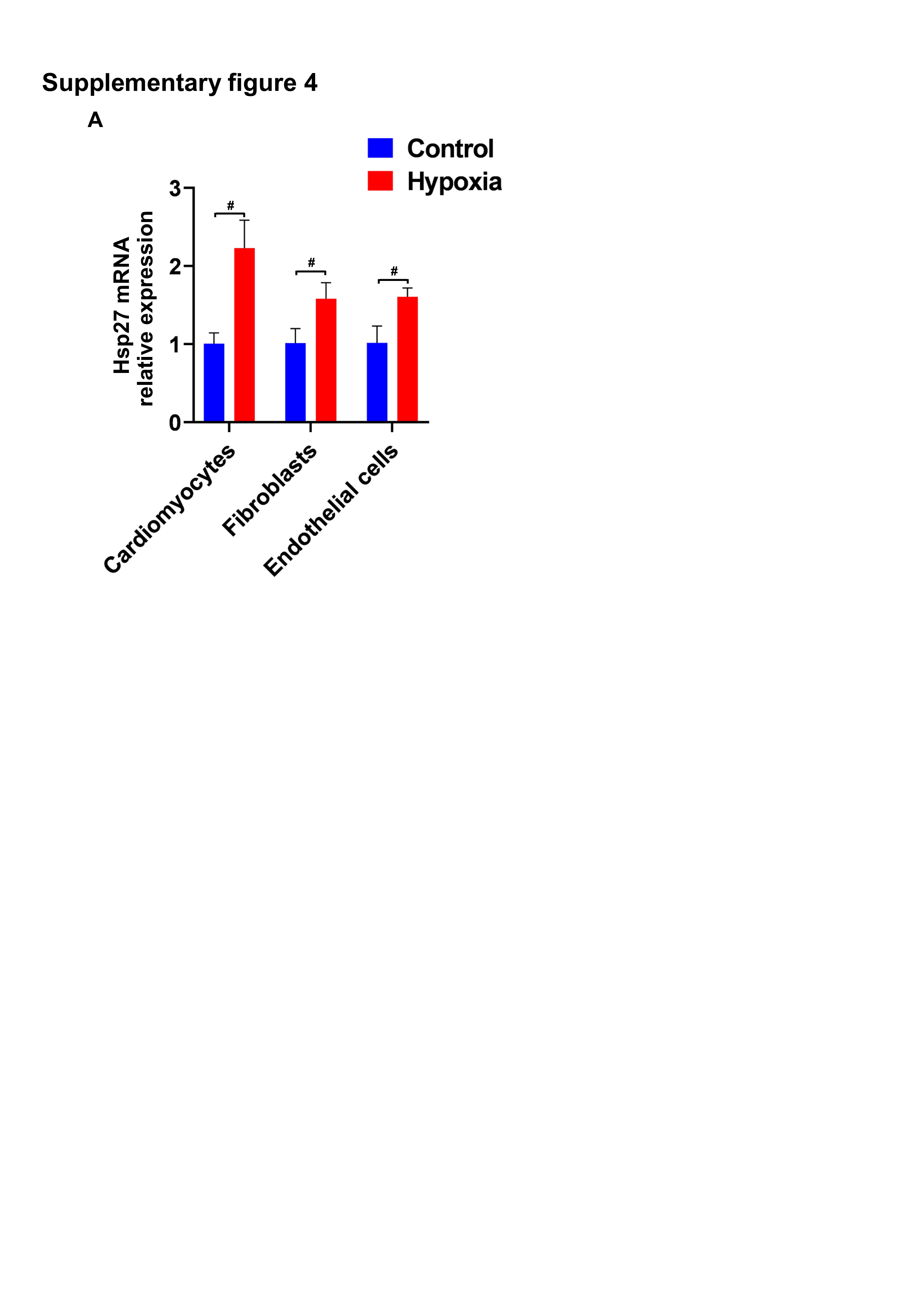


**Supplementary figure S4. The mRNA expression of *Hsp27* in different cells after treated with hypoxia**

(A) Relative mRNA level of *Hsp27* in cardiomyocytes, fibroblasts or endothelial cells induced by hypoxia for 12 h was determined by qRT-PCR. Data were represented by mean ± SD (n=6). ^#^*P* < 0.05 vs. Control group.


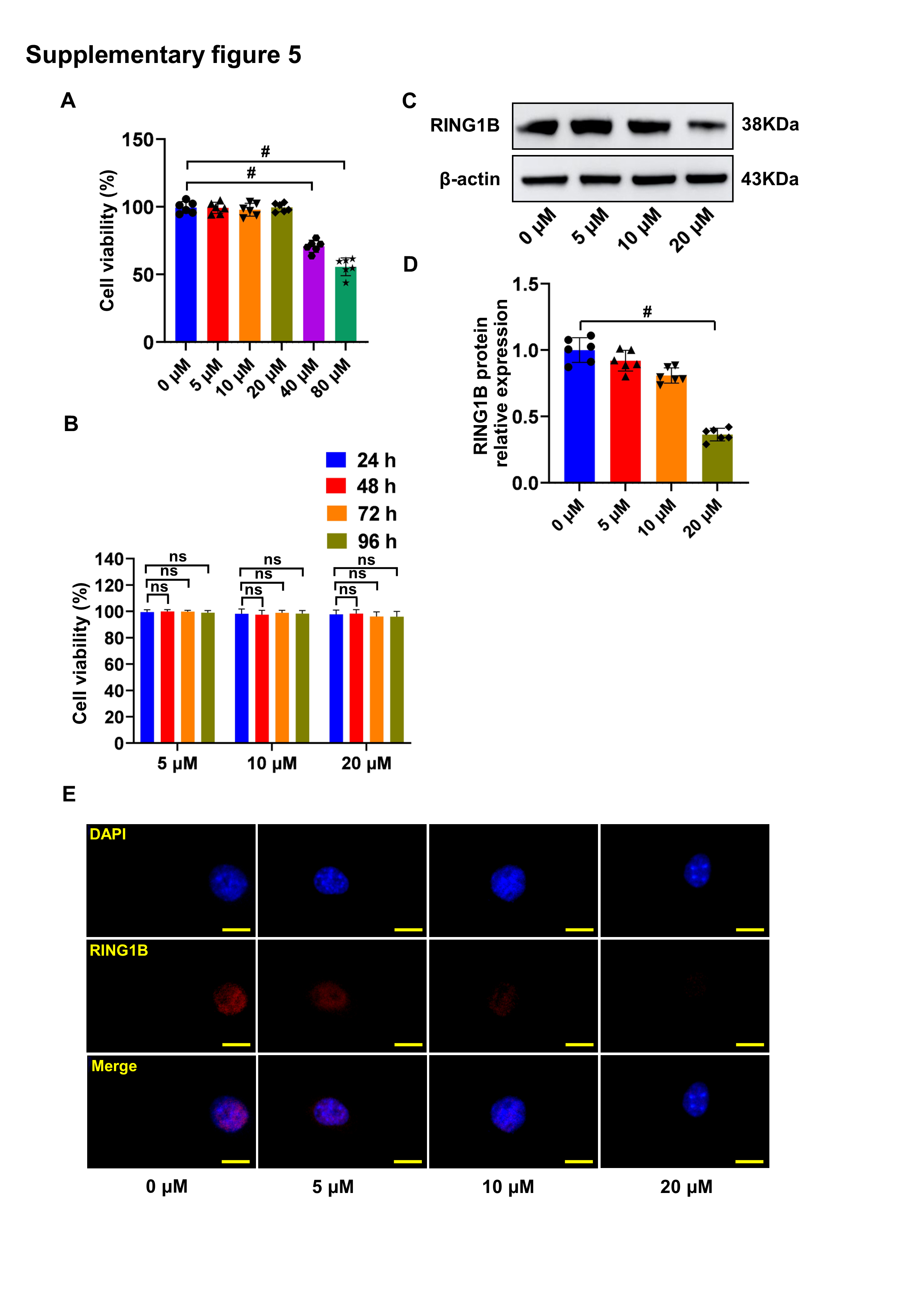


**Supplementary figure S5. PRT@PPMP inhibits the protein expression of *RING1B***

(A) Cell viability of cardiomyocytes was detected after treatment with PRT@PPMP at different doses. (B) Cell viability of cardiomyocytes was detected after treatment with PRT@PPMP at different time points. (C-D) *RING1B* protein expression level was determined by western blot in cardiomyocytes treated with PRT@PPMP at different doses. (E) Immunofluorescence of *RING1B* (red) staining in cardiomyocytes treated with PRT@PPMP at different doses. Data were represented by mean ± SD (n=6). ^#^*P* < 0.05 vs. 0 μM group. Scale bars, 25 μm. ns, not significant.


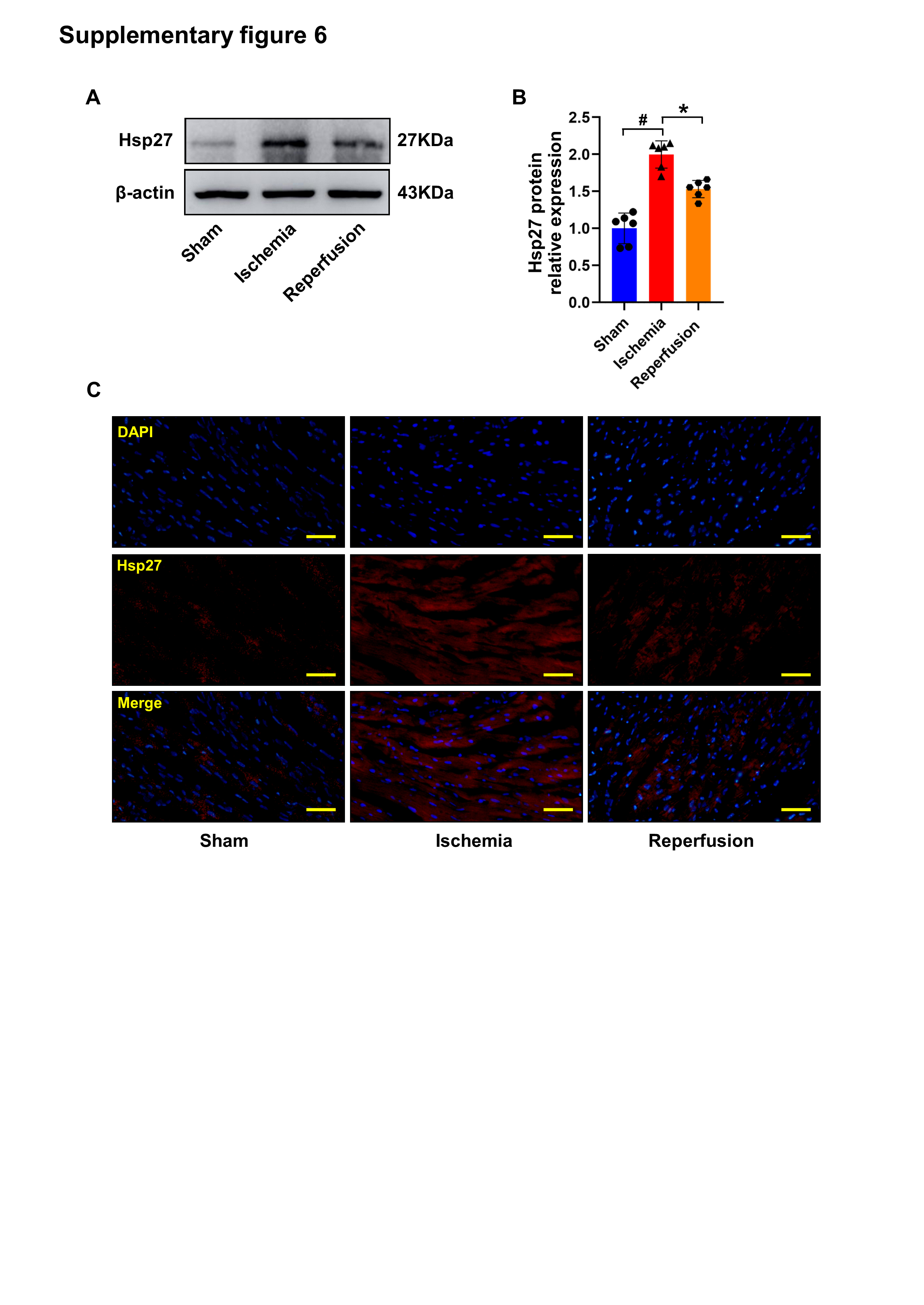


**Supplementary figure S6. *Hsp27*** **is decreased in a time-dependent manner during MI/RI**

(A-B) *Hsp27* expression in cardiac tissue was quantified by means of western blotting. (C) Representative images of *Hsp27* staining (red) in cardiac tissue of rats induced by MI/RI and counter-stained with DAPI (blue). Data were represented by mean ± SD (n=6). ^#^*P* < 0.05 vs. Sham group; ^*^*P* < 0.05 vs. Ischemia group. Scale bars, 50 μm.


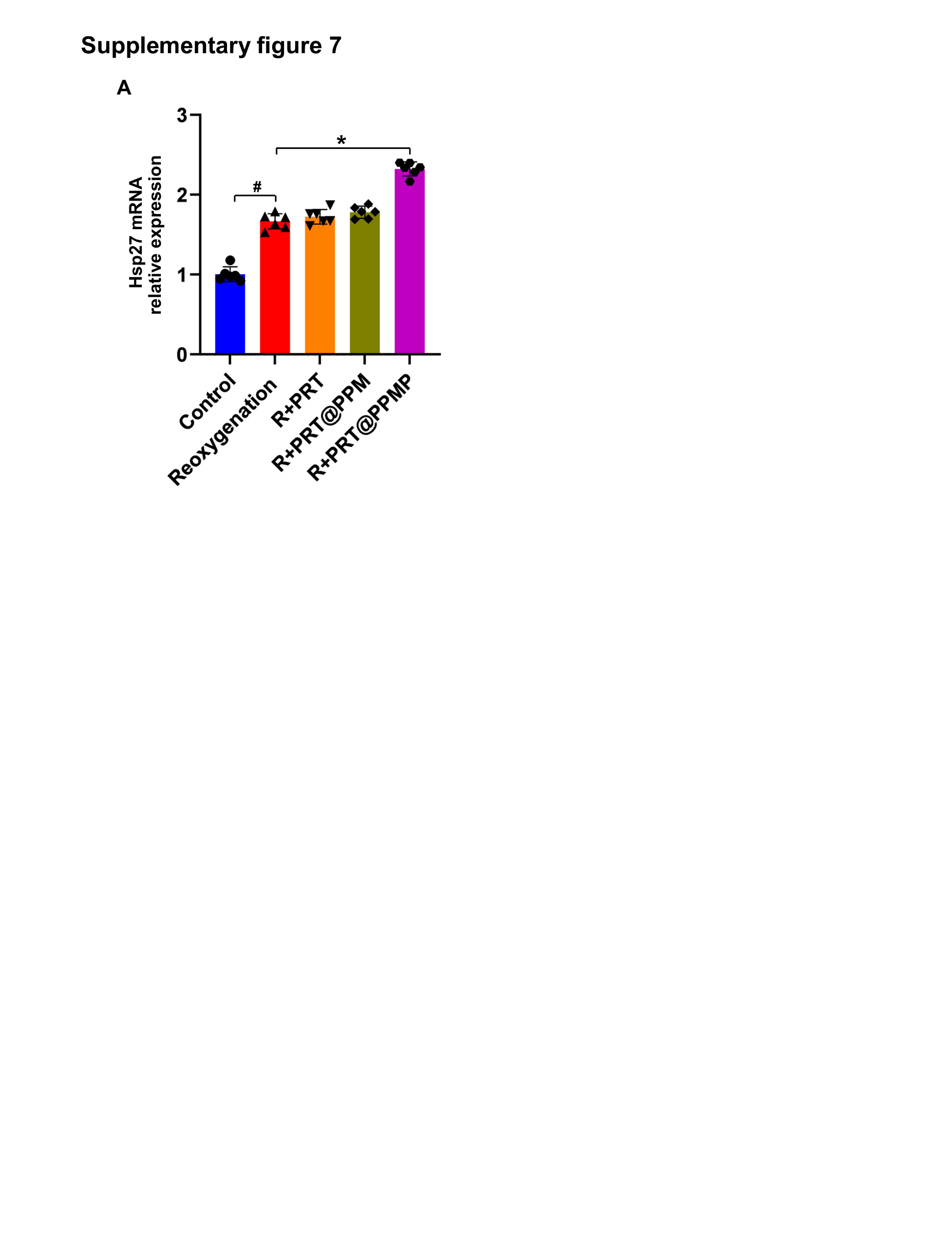


**Supplementary figure S7. PRT@PPMP up-regulates the mRNA expression of *Hsp27***

(A) The mRNA expression of *Hsp27* in H/R-induced cardiomyocytes was measured by qRT-PCR after treated with PRT, PRT@PPM and PRT@PPMP, respectively. Data were represented by mean ± SD (n=6). ^#^*P* < 0.05 vs. Control group; ^*^*P* < 0.05 vs. Reoxygenation group.


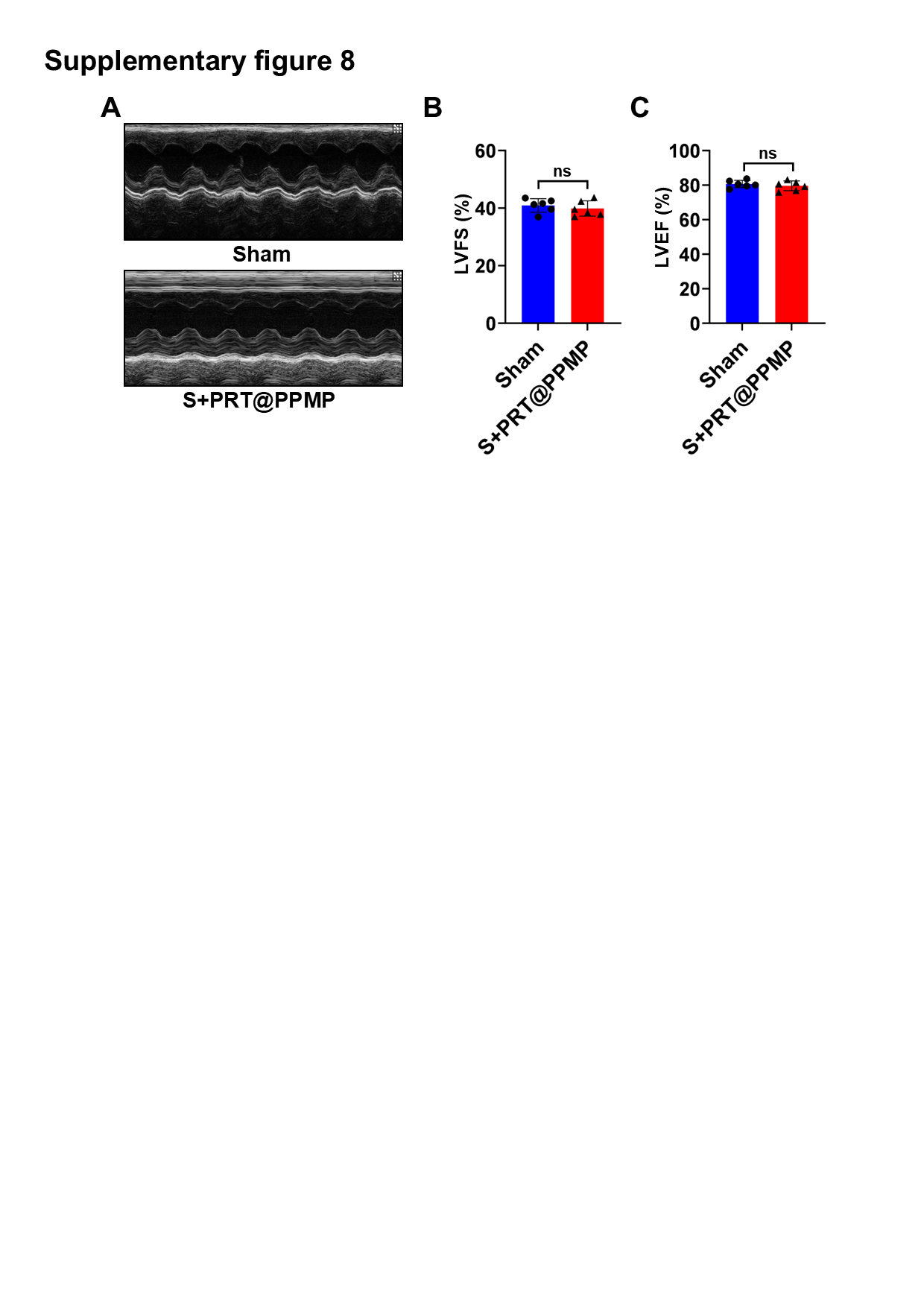


**Supplementary figure S8. PRT@PPMP has no effect on normal heart function**

(A) Representative echocardiography images. (B-C) The echocardiographic parameters including left ventricular fractional shortening (LVFS) and left ventricular ejection fraction (LVEF) were measured in sham rats treated with or without PRT@PPMP. S+PRT@PPMP group: Sham rats treated with PRT@PPMP. Data were represented by mean ± SD (n=6). ns, not significant.


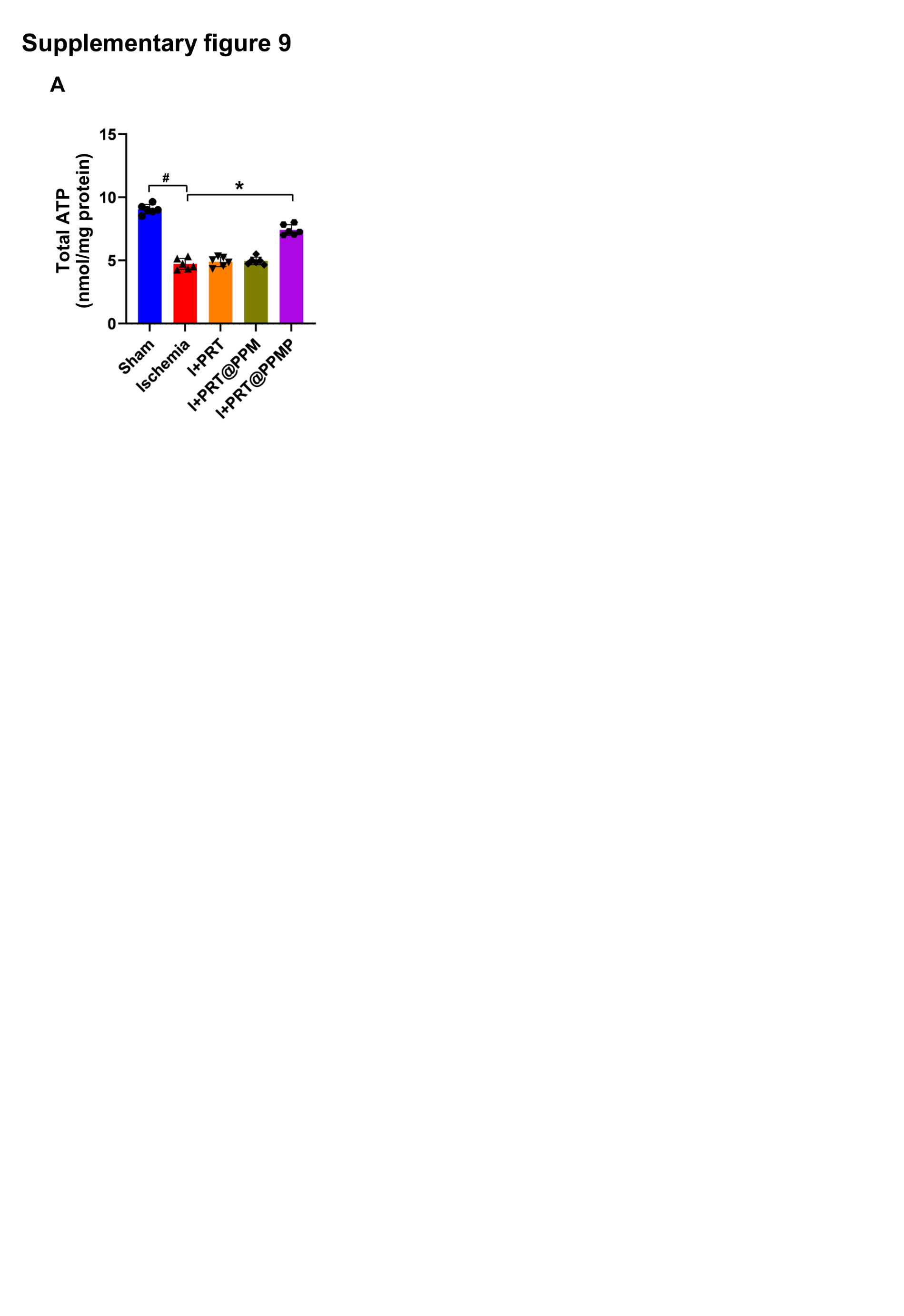


**Supplementary figure S9. PRT@PPMP increases the ATP level in ischemia hearts**

(A) ATP levels were detected in myocardial tissue of ischemia rats. Data were represented by mean ± SD (n=6). ^#^*P* < 0.05 vs. Sham group; ^*^*P* < 0.05 vs. Ischemia group.

**Table S1. Primer sequence**

| **Name** | **Forward (5'-3')** | **Reverse (5'-3')** |
| --- | --- | --- |
| ptgs2 | CAACCAGCAGTTCCAGTATCAG | GAGCAAGTCCGTGTTCAAGG |
| Il1b | GGGATGATGACGACCTGCTA | TGTCGTTGCTTGTCTCTCCT |
| Myc | CATCAAGAGGCCACAGCAAA | GCAGCTGGATAGTCCTTCCT |
| Hspa1b | AAGAATGCGCTCGAGTCCTA | GATCTTGCCCTTGAGACCCT |
| Pgf | TCTCTCAGGATGTGCTCTGC | TTGCTTTGCTTCCTCTTCCC |
| Cd14 | CTTATGCTCGGCTTGTTGCT | CAGTAGCAGCGGACACTTTC |
| Map3k6 | CAGGACTACTCCGCCATCAT | ACGTGTAGTGGAAGCAGACA |
| Dusp2 | TCCGCTACAAGAGCATTCCA | ATGAAGCCGATAGCCTCCTG |
| Hsp27 | GGTGCTTCACCCGGAAATAC | CTCCGCTGATTGTGTGACTG |
| Flnc | TCCACCGATGTGTCACTGAA | CTCATTGCCTGAAGGAGCAC |
| Il1a | GCTCTTTGTGAGTGCTCAGG | GAAAGCTGCGGATGTGAAGT |
| PFKFB3 | CTTCTTCCGCCCTGACAATG | GCCTCCTTTCTCTCGTCGTA |
| HK3 | TGTGAGATGGGCCTCATTGT | GCCACATTCCGGAGTTCTTC |
| TP53 | GGAGTGCAAAGAGAGCACTG | CAGCACGGGCATCCTTTAAT |
| PFKL | GTACGTTGTGCATGATGGCT | TCTACAATGGCCTCCAGGTG |
| COQ9 | TTCCTGGACAACCGGATCAA | CAGGTTCTTGAGCGTGACTG |

**Table S2. Primary antibodies information**

| **Proteintech group** | **Catalog number** | **Name** | **Dilution ratio** |
| --- | --- | --- | --- |
| ZSGB-BIO | TA-09 | β-actin | 1:2000 |
| Cell Signaling Technology | #8240 | H2Aub | 1:1000 |
| Santa Cruz Biotechnology | sc-13132 | Hsp27(IP) | 1:50 |
| Santa Cruz Biotechnology | sc-365073 | COQ9 | 1:1000 |
| ABclonal | A5563 | RING1B | 1:1000 |
| ABclonal | A11156 | Hsp27(IF) | 1:100 |
| ABclonal | A16926 | NDUFS1 | 1:1000 |
| ABclonal | A6585 | ETFDH | 1:1000 |
| ABclonal | A7670 | ETFA | 1:1000 |
| ABclonal | A14752 | NDUFB5 | 1:1000 |

**Table S3. Primer information of knockdown genes**

| **Usage** | **Name** | **Primer Sequence 5'-3'** |
| --- | --- | --- |
| Knockdown genes | Il1b | CAGGCUUCGAGAUGAACAATT;  UUGUUCAUCUCGAAGCCUGTT |
| Knockdown genes | Myc | GCGACGAGGAAGAGAAUUUTT;  AAAUUCUCUUCCUCGUCGCTT |
| Knockdown genes | Hspa1b | CCUACGCCUUCAAUAUGAATT;  UUCAUAUUGAAGGCGUAGGTT |
| Knockdown genes | Pgf | GGUGUACAUUGCAGAUGAATT;  UUCAUCUGCAAUGUACACCTT |
| Knockdown genes | Cd14 | GCCUGGAGUACCUUCUAAATT;  UUUAGAAGGUACUCCAGGCTT |
| Knockdown genes | Map3k6 | GCCUUCGCCACAAGAAUAUTT;  AUAUUCUUGUGGCGAAGGCTT |
| Knockdown genes | Dusp2 | CAUUCCAGUAGAAGAUAAUTT;  AUUAUCUUCUACUGGAAUGTT |
| Knockdown genes | Hsp27 | GCCUCUUCGAUCAAGCUUUTT;  AAAGCUUGAUCGAAGAGGCTT |
| Knockdown genes | Flnc | GCCCUUUCAAGAUCAAAGUTT;  ACUUUGAUCUUGAAAGGGCTT |
| Knockdown genes | Il1a | GAGGAGACGACUCUAAAUATT;  UAUUUAGAGUCGUCUCCUCTT |
| Knockdown genes | PFKFB3 | GGAAGGCGCUCAAUGAGAUTT;  AUCUCAUUGAGCGCCUUCCTT |
