# Supplementary material for: Upregulation of Hsp27 via further inhibition of histone H2A ubiquitination confers protection against myocardial ischemia/reperfusion injury by promoting glycolysis and enhancing mitochondrial function
Source: Cell Death Discov. 2023 Dec 19;9:466. doi: 10.1038/s41420-023-01762-x (PMC10730859; doi:10.1038/s41420-023-01762-x)
Supplement: Supplementary file 2 — Original western blots [file 41420_2023_1762_MOESM2_ESM.docx]

**Fig. 2I**

**
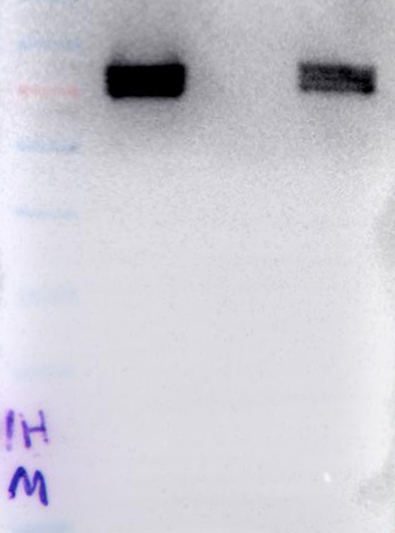

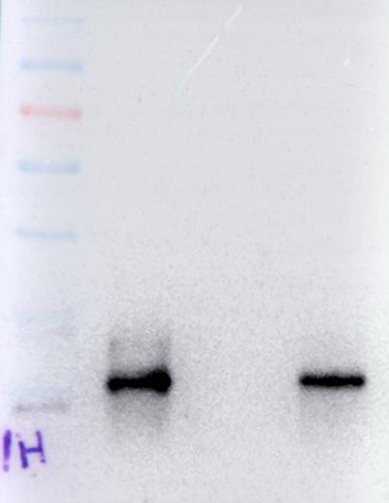
**

**84KDa**

**27KDa**

**p-IKKα**

**Hsp27**

**Fig. 4A**

**
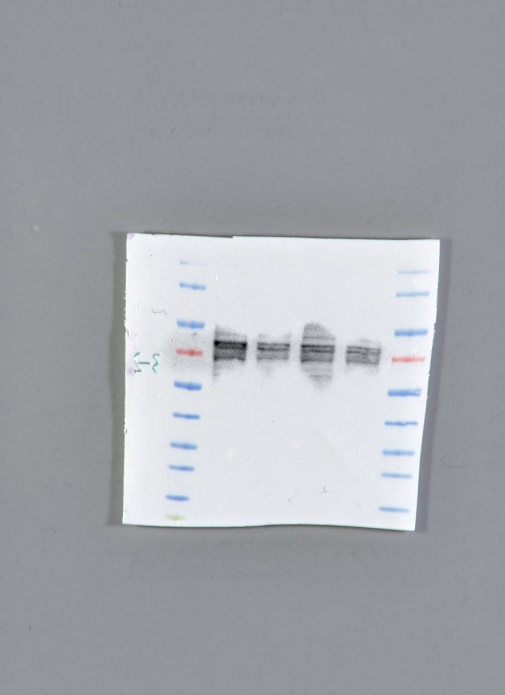

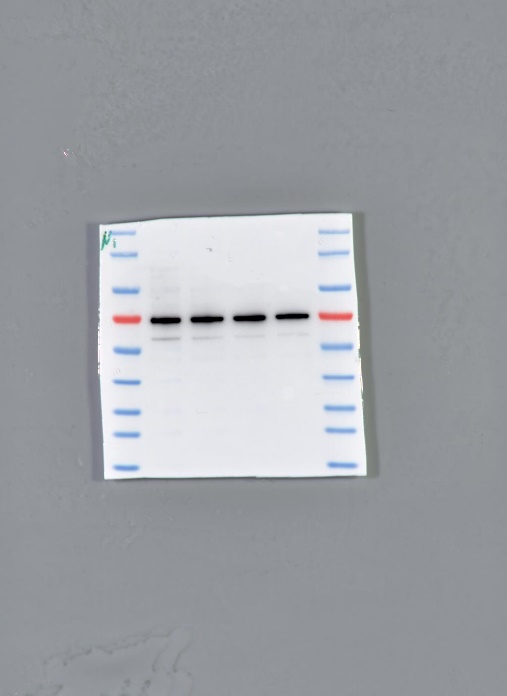
**

**75KDa**

**65KDa**

**NDUFS1**

**ETFDH**


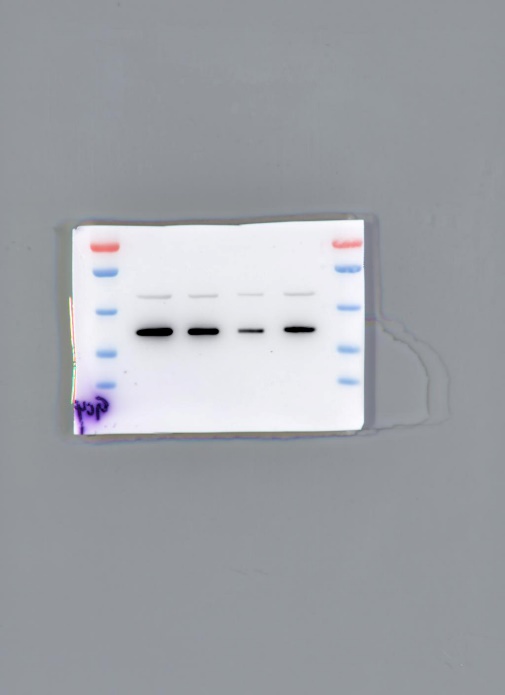

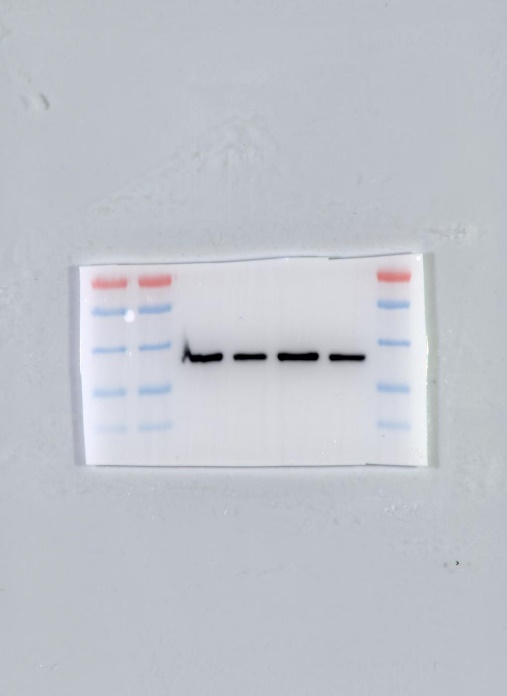


**36KDa**

**35KDa**

**ETFA**

**COQ9**


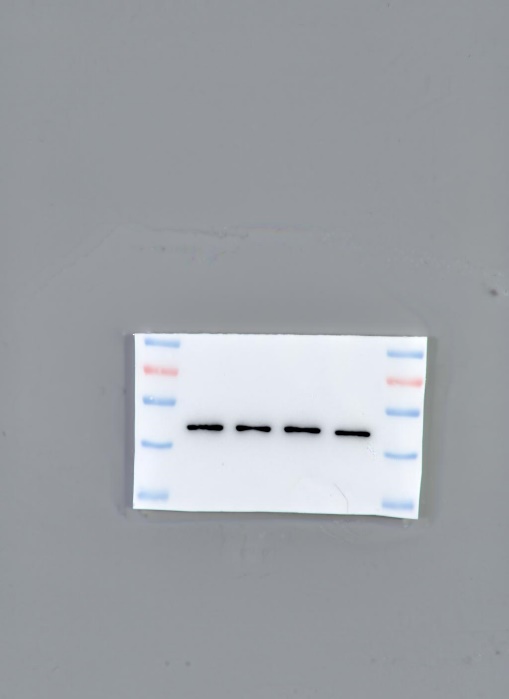

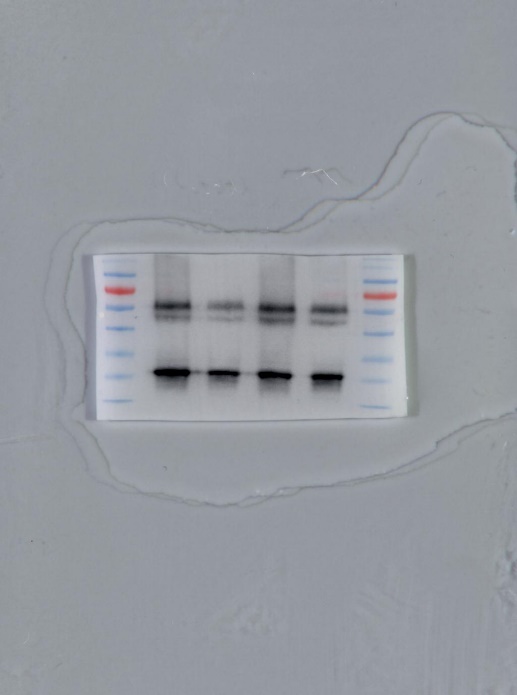


**43KDa**

**NDUFB5**

**22KDa**

**β-actin**

**Fig. 4D**


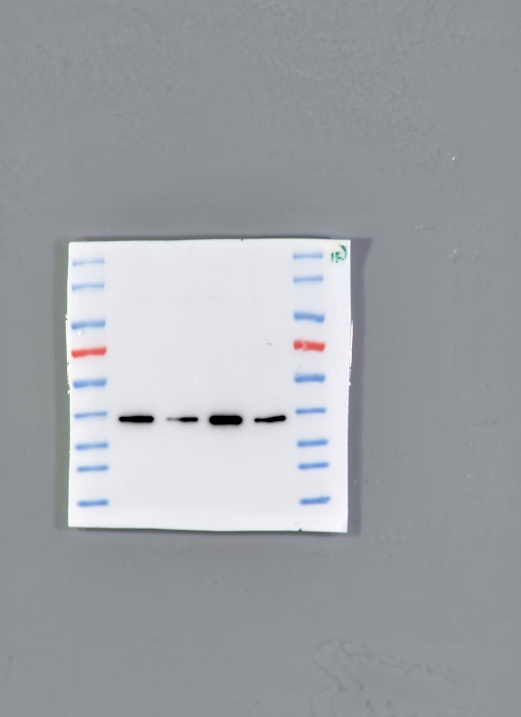

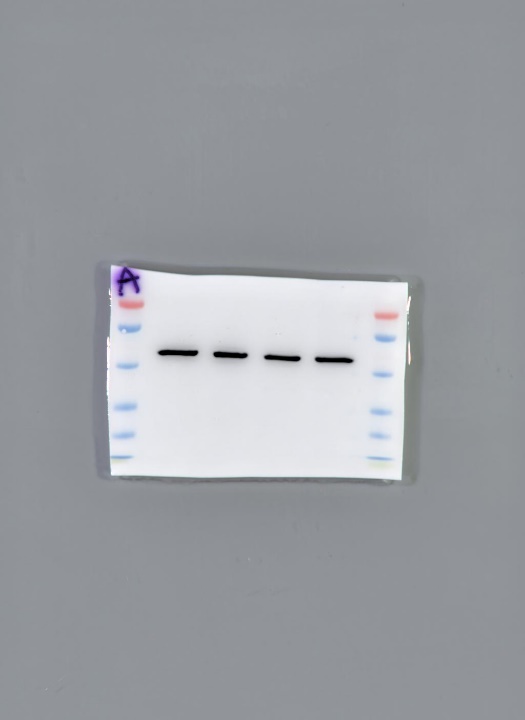


**β-actin**

**43KDa**

**36KDa**

**COQ9**

**Fig. 4I**

**36KDa**


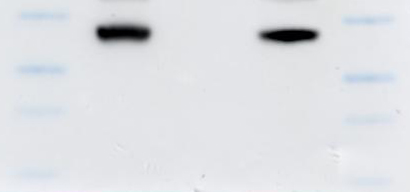

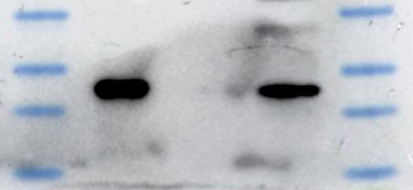


**27KDa**

**COQ9**

**Hsp27**

**Fig. 5G**


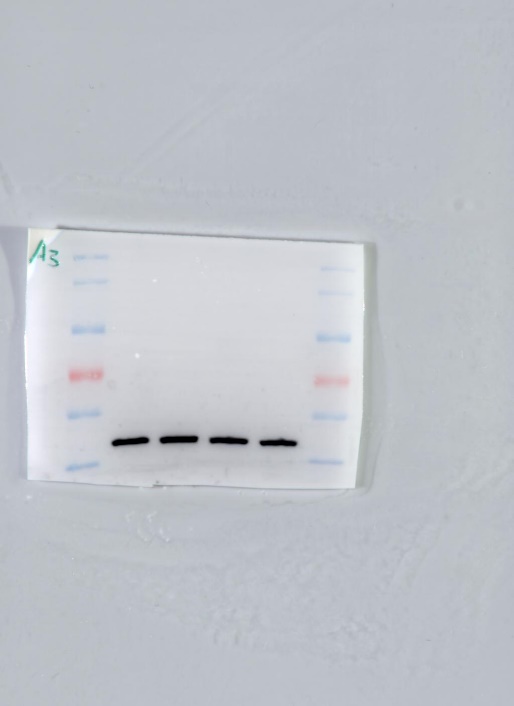

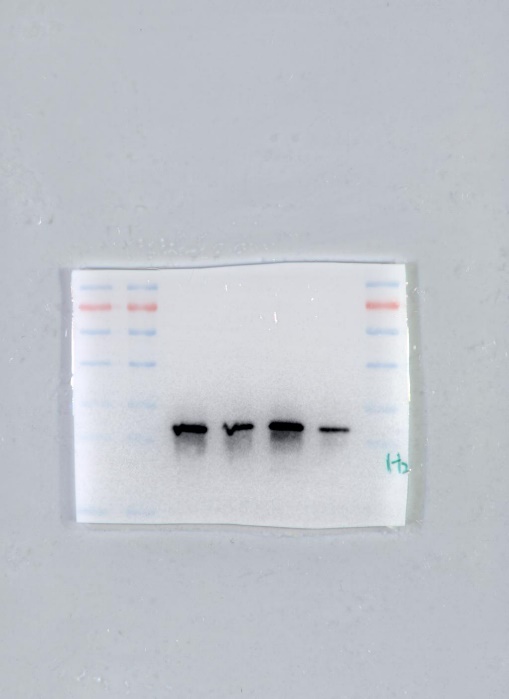


**23KDa**

**43KDa**

**H2Aub**

**β-actin**

**Supplementary** **Fig. 5C**


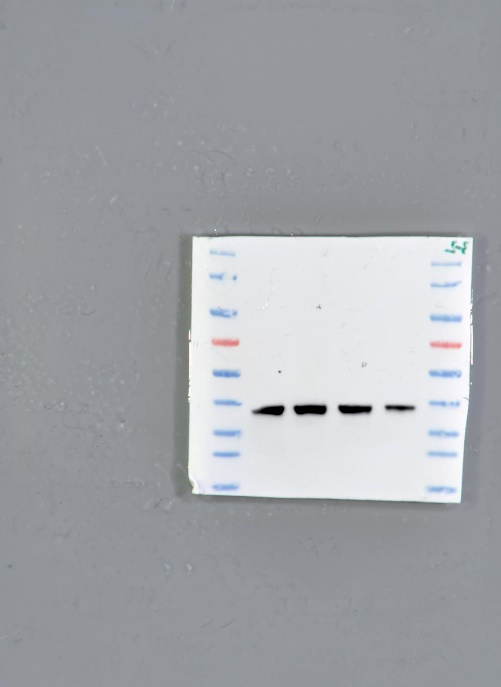

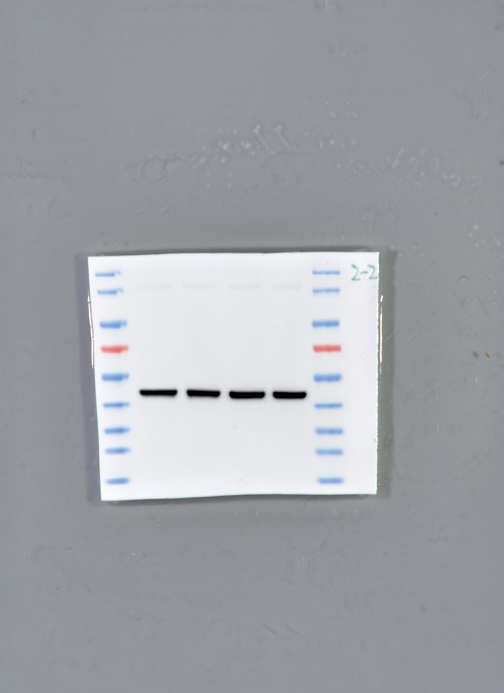


**38KDa**

**43KDa**

**RING1B**

**β-actin**

**Supplementary** **Fig. 6A**


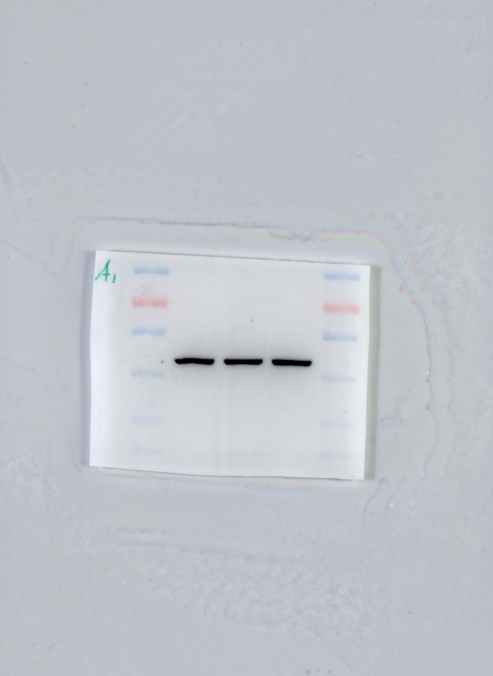

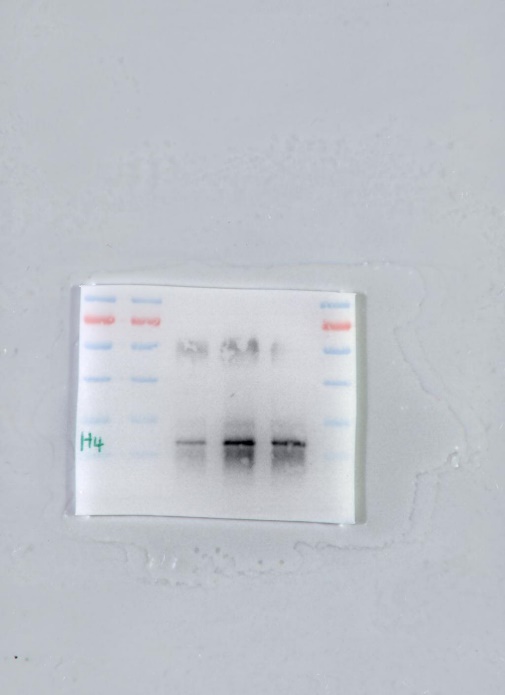


**43KDa**

**27KDa**

**β-actin**

**Hsp27**
